# Global Burden of Acne (1990–2021) and Projections for China's Disease Burden Over the Next 15 Years: A Comprehensive Study Based on Global Burden of Disease Data

**DOI:** 10.1111/jocd.71118

**Published:** 2026-09-25

**Authors:** Qian Zhang, Peixuan Wu, Chun Yang, Zijie He, Yanyan Feng

**Affiliations:** ^1^ Department of Dermatology, Sichuan University Affiliated Chengdu Second People's Hospital, Chengdu Second People's Hospital, West China School of Medicine Sichuan University Chengdu China

## Abstract

**Background:**

Acne vulgaris is a common chronic inflammatory skin disease with a significant impact on the global health burden. Although acne vulgaris is more prevalent among adolescents, the incidence of adult acne is gradually increasing, imposing a severe psychological and social burden, particularly on women of reproductive age. China accounts for 18% of the global population, yet previous GBD studies have paid insufficient attention to the evolving burden of acne in the country. With China's Socio‐demographic Index (SDI) rising rapidly, it serves as an ideal case for examining the relationship between socioeconomic transition and disease burden. This study aims to analyze trends in the global burden of acne vulgaris from 1990 to 2021, examine regional, national, age, and gender disparities, assess associations with sociodemographic indicators, and project future trends in China's acne vulgaris burden over the next 15 years. Prior studies have largely been confined to single continents or specific countries, with data typically limited to 2017 or earlier. In contrast, this study spans 204 countries and territories worldwide from 1990 to 2021, significantly expanding both temporal and spatial coverage. It is the first to systematically delineate nonlinear associations within the Socio‐demographic Index (SDI) framework, revealing a distinctive epidemiological curve characterized by an initial decline followed by a subsequent rise. Furthermore, by incorporating projections of China's disease burden over the next 15 years, this research provides robust empirical evidence to inform public health policymaking across the Asia‐Pacific region.

**Methods:**

Using the Global Burden of Disease (GBD) 2021 database, we extracted data on acne vulgaris incidence, prevalence, disability‐adjusted life years (DALYs), and age‐standardized rates from 204 countries and regions worldwide between 1990 and 2021. Trend analysis employed age‐standardized rates, estimated annual percentage change (EAPC), and Bayesian modeling, with socioeconomic factors assessed using the Socio‐Demographic Index (SDI).

**Results:**

In 2021, 239.7 million prevalent cases and 124.3 million incident cases of acne vulgaris were reported globally, accounting for 5.126 million DALYs. The global age‐standardized prevalence, incidence, and DALY rates for acne vulgaris were 3142.5 per 100 000 population (95% uncertainty interval: 2867.0 to 3500.6), 1645.2 (1459.7 to 1878.2), and 67.2 (42.3 to 105.8) per 100 000 population, representing increases of 12.2%, 14%, and 12.3% compared to 1990. In 2021, Germany (5688.4), Portugal (5475.1), and Luxembourg (5323.1) exhibited the highest age‐standardized prevalence rates for acne vulgaris. Over the entire study period, Sudan (25.3), Equatorial Guinea (25.2), Iraq (24.9), and Oman (24.7) recorded the largest increases in age‐standardized point prevalence rates. In 2021, Germany (2723) and Romania (821.7) had the highest and lowest age‐standardized incidence rates, respectively, while Germany (121) and Romania (32.2) had the highest and lowest age‐standardized DALY rates, respectively. Gender differences were pronounced, with women exhibiting higher standardized prevalence, incidence, and DALY rates than men. Among men, the global DALY rate for acne vulgaris increased between ages 15–19 and then declined with age, while for women, this rate also rose between ages 15–19. Regionally, a complex association exists between sociodemographic indices and the age‐standardized DALY rate for acne vulgaris, characterized by an overall plateau followed by a steep upward trajectory. China's burden of acne vulgaris exceeds the global average and is projected to continue rising over the next 15 years.

**Conclusion:**

Acne vulgaris represents a significant global dermatological burden, particularly in high‐SDI regions, among children, and in female populations. Targeted prevention and treatment strategies are needed to mitigate its health impacts.

## 1 Introduction

Acne vulgaris is a common chronic inflammatory disease of the follicular‐sebaceous unit, primarily affecting sebum‐rich areas such as the face, chest, and back. Clinical manifestations include pimples, papules, pustules, cysts, and nodules [1]. Approximately 9.4% of the global population is affected by acne vulgaris, making it the fourth leading cause of non‐fatal disease burden worldwide and the eighth most prevalent disease [2, 3, 4]. Acne vulgaris not only causes pain and itching but may also lead to scarring and psychological issues such as anxiety, depression, and social avoidance, significantly reducing patients' quality of life [5, 6]. In recent years, the prevalence of adult acne vulgaris (aged 25+) has significantly increased, particularly among women of reproductive age (15–49), a trend closely linked to hormonal changes, stress, diet, and environmental factors [7, 8]. The Global Burden of Disease (GBD) study provides a comprehensive tool for assessing health loss, quantifying both non‐fatal and fatal burdens through disability‐adjusted life years (DALYs). Previous studies indicate skin diseases rank as the fourth leading cause of non‐fatal burden globally, with acne vulgaris contributing significantly [9]. However, while studies have examined acne vulgaris burden in Asia, Europe, or specific countries, comprehensive global analyses spanning 1990–2021—particularly tracking dynamic changes within the Socio‐Demographic Index (SDI) framework—remain scarce [9, 10, 11]. As a composite indicator of socioeconomic development encompassing income, education, and social factors, SDI's association with acne vulgaris burden may reveal underlying mechanisms of health inequities [12]. For instance, higher SDI regions may exhibit visible burden due to increased diagnostic rates, while lower SDI regions may conceal burden due to environmental exposures and poor healthcare accessibility [13, 14]. This study systematically analyzes global changes in the burden of acne vulgaris from 1990 to 2021 using GBD 2021 data, with a particular focus on the Chinese context, aiming to inform public health policy.

## 2 Methods

### 2.1 Data Sources

Data were obtained from the publicly available GBD 2021 database (http://ghdx.healthdata.org/gbd‐2021), covering prevalence, incidence, and DALYs for 371 diseases and injuries across 204 countries and regions [15]. Acne vulgaris is classified as a noncommunicable disease within the GBD 2021 hierarchy, specifically under diseases of the skin and subcutaneous tissue. The definition of acne vulgaris is based on International Classification of Diseases, 10th Revision (ICD‐10) code L70 and ICD‐11 code ED80 [8]. Data extraction includes global, regional, and national levels of acne vulgaris prevalence, incidence, DALYs, and corresponding age‐standardized rates (age‐standardized prevalence rate [ASPR], age‐standardized incidence rate [ASIR], age‐standardized DALY rate [ASDR]) for 1990–2021, stratified by age, sex, and socio‐demographic index (SDI). SDI data sourced from GBD ranges from 0 (lowest development level) to 1 (highest development level), calculated as a composite indicator based on per capita lagged distributed income, mean years of schooling, and total fertility rate under age 25 [16]. Spatial analysis visually displays country‐level disparities through maps, while age and sex stratification employs 5‐year age groups.

### 2.2 Statistical Analysis

Age standardization using the GBD world standard population to eliminate differences in population structure. A smoothed spline model was used to examine the relationship between acne burden (DALYs) and the Sociodemographic Index (SDI) across 21 regions and 204 countries and territories. Predictive Analysis: Predicts trends in the incidence of acne vulgaris in China from 2022 to 2036 using a Bayesian age‐period‐cohort (BAPC) model. Statistical Software: Data analysis performed using R 4.3.3 with a significance level set at *p* < 0.05.

## 3 Results

### 3.1 Global Level

In 2021, 239.7 million prevalent cases of acne vulgaris were reported globally, with an age‐standardized point prevalence of 3142.5 per 100 000 population, representing a 12.2% increase since 1990. In 2021, acne vulgaris caused 124.3 million cases, with an age‐standardized incidence rate of 1645.2 per 100 000 population, representing a 14.0% increase since 1990. In 2021, global acne vulgaris DALYs totaled 5.126 million, with an age‐standardized rate of 67.2 per 100 000 population, representing a 12.3% increase since 1990 (Table 1).

**TABLE 1:** Prevalence, incidence, and disability‐adjusted life years (DALYs) for acne vulgaris in 2021, by global burden of disease region, and percentage change in age‐standardized rates (ASRs) per 100 000 population from 1990 to 2021.

Location	Prevalence No_in_millions_95% UI	ASPRs_per_100 000_95% UI	Percentage_change_in_ASPRs_from_1990_to_2021	Incidence No_in_thousands_95% UI	ASIRs per 100 000 95% UI	Percentage change in ASIRs from 1990 to 2021	DLAYs. Number in thousands 95% UI	ASDRs per 100 000 95% UI	Percentage change in ASDRs from 1990 to 2021
Global	239.7 (219, 266.5)	3142.5 (2867, 3500.6)	12.2 (11.2, 13.2)	124 266.4 (110, 490.2, 141 552)	1645.2 (1459.7, 1878.2)	14 (12.6, 15.4)	5125.5 (3225, 8069.8)	67.2 (42.3, 105.8)	12.3 (11.2, 13.5)
High‐income Asia Pacific	5.4 (5, 5.8)	4307 (3949.1, 4760.3)	8.3 (6.5, 10.3)	2576.2 (2340.3, 2849.7)	2220.3 (1967.8, 2514.6)	8.8 (6.6, 10.8)	114.3 (71.6, 178.6)	92.3 (57.8, 143.8)	8.4 (5.9, 10.9)
High‐income North America	9.8 (9.5, 10.3)	3103.7 (2977.7, 3263.1)	8.9 (5.3, 13.1)	4868.4 (4611.6, 5155.6)	1605.5 (1511.7, 1714.1)	12.8 (8.1, 18.6)	207.1 (129.4, 327.5)	65.6 (41, 103.5)	8.2 (4.2, 13.4)
Western Europe	16.8 (15.6, 18.3)	5154.7 (4726.6, 5685.4)	7.5 (6.9, 9.3)	8014.7 (7193.8, 8991)	2583.9 (2278.4, 2947.5)	8.6 (6.5, 10.8)	357.1 (223.6, 556.7)	109.7 (69, 171.3)	7.4 (5.3, 9.6)
Australasia	0.9 (0.8, 1)	3437.7 (3098.6, 3874.2)	7.6 (3.4, 11.6)	448.3 (398.2, 510.7)	1822.1 (1598.6, 2104.6)	7.2 (1.9, 12)	18.5 (11.4, 28.9)	73.1 (45.4, 114.3)	7.6 (1.7, 14.8)
Andean Latin America	2.4 (2.1, 2.7)	3497.6 (3125.6, 3941.7)	6.9 (2.4, 10.9)	1244.2 (1057.8, 1453.5)	1878.7 (1590.1, 2201.1)	7.4 (1.7, 12.6)	50.7 (30.8, 79.9)	75.1 (45.6, 118.5)	7.1 (1.3, 13.1)
Tropical Latin America	4.7 (4.3, 5.3)	2341.9 (2104.4, 2638.6)	10.9 (9.3, 12.5)	2455.1 (2167.4, 2802.9)	1265.2 (1108.2, 1454.5)	11.6 (9.8, 13.6)	100.6 (62.5, 159.6)	50 (31, 79.3)	11 (8.3, 13.5)
Central Latin America	6.5 (5.8, 7.3)	2510.4 (2243.1, 2858.3)	11.6 (10.1, 13.3)	3380.4 (2945.3, 3926.6)	1349.1 (1174.3, 1574.3)	12.1 (10.3, 14.1)	138.7 (85.3, 218.7)	53.9 (33.2, 85)	11.8 (9.3, 14.5)
Southern Latin America	2.2 (2, 2.5)	3467.3 (3099.9, 3918.3)	9 (5.6, 12.2)	1123.2 (991.2, 1279.1)	1799.4 (1573.4, 2068.4)	9.1 (4.9, 12.8)	47.4 (29, 74.7)	73.8 (45, 116.5)	8.8 (3, 14.5)
Caribbean	1.1 (1, 1.3)	2547.1 (2243.4, 2938.4)	10.9 (7.9, 14.3)	601.3 (511.9, 714.3)	1373.1 (1164.9, 1639.1)	11.7 (8.1, 15.7)	24.5 (15, 38.7)	54.4 (33.3, 85.8)	10.9 (6.6, 15.9)
Central Europe	1.2 (1.1, 1.3)	1564.7 (1460, 1684.2)	10.7 (8.3, 13.3)	637.2 (591.0, 692.2)	838.8 (773.6, 916.9)	12.2 (9.6, 15.3)	26.2 (16.3, 41.2)	33.6 (20.8, 52.9)	10.8 (7.4, 14.8)
Eastern Europe	2.7 (2.4, 3)	1875 (1686.8, 2112.3)	9.2 (8.0, 10.7)	1407.8 (1251.5, 1596.4)	983.7 (870.2, 1119.4)	9.3 (7.9, 10.8)	57.6 (36, 91.7)	40.2 (24.9, 64.1)	9.2 (6.9, 11.8)
Central Asia	1.6 (1.4, 1.9)	1822.5 (1604, 2084.7)	9.1 (6.4, 11.7)	867.5 (748.6, 1007.7)	959.5 (828, 1114.2)	9.2 (6.6, 12.3)	35.1 (21.8, 55.5)	39.2 (24.4, 62)	9.3 (4.5, 13.7)
North Africa and Middle East	20.2 (18.0, 22.6)	3102.5 (2773.7, 3487.6)	20.6 (18.8, 22.6)	10740.2 (9334.1, 12447.1)	1646 (1430.2, 1906)	21.6 (19.4, 24)	431.3 (268.5, 678.6)	66.4 (41.4, 104.4)	20.6 (17.9, 23.2)
South Asia	48.2 (43.5, 54.3)	2334.3 (2109, 2626.6)	18.8 (17.5, 20)	24632.5 (21749.5, 28001.2)	1217.1 (1071.6, 1387)	18.9 (17.3, 20.3)	1029.4 (643, 1633.1)	49.9 (31.2, 79.1)	19.3 (17.2, 21.4)
Southeast Asia	22.6 (20.4, 25.4)	3285.1 (2956.7, 3695.9)	14 (12.7, 15.4)	11410.8 (10 110, 13058.7)	1694.5 (1492.8, 1947.6)	13.8 (12.6, 15.2)	485.5 (305.5, 769.2)	70.6 (44.4, 111.9)	14.3 (12.3, 16.2)
East Asia	40.9 (37.4, 44.8)	3738.6 (3376.3, 4150.5)	18 (16.2, 19.7)	20573.8 (18352.4, 23253.3)	1913.7 (1691.1, 2181.1)	17.3 (15.5, 19)	879.6 (549.7, 1377.7)	80.7 (50.5, 126.5)	18.3 (16, 20.8)
Oceania	0.4 (0.4, 0.5)	2709.9 (2398.5, 3098.5)	9.6 (6.5, 13.2)	230.9 (198.0, 269.3)	1454.1 (1253.8, 1688.5)	10.1 (6.5, 14.6)	9.1 (5.6, 14.5)	58.1 (36.1, 92.7)	9.7 (3.6, 15.9)
Western Sub‐Saharan Africa	20.4 (18.1, 22.9)	3283.1 (2945.6, 3666.2)	15.4 (14.2, 16.8)	11 504 (9845.3, 13440.6)	1775.9 (1534.1, 2051.3)	16.5 (14.8, 18.3)	435.1 (270.6, 687)	70.1 (43.6, 110.6)	16 (14.3, 17.8)
Eastern Sub‐Saharan Africa	22.7 (20.5, 25.5)	4084.6 (3703.4, 4552.2)	14.2 (12.8, 15.9)	12589.3 (10761.6, 14633.4)	2188.7 (1891.0, 2526.1)	15.3 (13.5, 17.3)	486.1 (301.6, 767.1)	87.2 (54.2, 137.5)	14.8 (12.9, 17.1)
Central Sub‐Saharan Africa	5.8 (5.1, 6.8)	3381.6 (2994.9, 3880.3)	13.1 (8.6, 18.5)	3296.1 (2748.4, 3968)	1821.6 (1538.1, 2167.2)	14.6 (9.2, 21.5)	124.8 (78.2, 197.6)	72.1 (45.1, 113.8)	14.1 (7.9, 21.3)
Southern Sub‐Saharan Africa	3.1 (2.8, 3.5)	3603 (3261.7, 4061.1)	12.6 (11.0, 14.7)	1664.5 (1444.8, 1942.5)	1912 (1661.6, 2230.1)	13.5 (11.3, 16.4)	66.8 (41, 105.5)	76.9 (47.2, 121.4)	12.5 (9.7, 15.6)

### 3.2 Regional Levels

In 2021, the highest age‐standardized prevalence rates of acne vulgaris per 100 000 population were observed in Western Europe (5154.7), high‐income Asia‐Pacific (4307.0), and Eastern Sub‐Saharan Africa (4084.6). Conversely, the lowest rates were found in Central Europe (1564.7), Central Asia (1822.5), and Eastern Europe (1875.0) had the lowest age‐standardized prevalence rates. In 2021, the highest age‐standardized incidence rates for acne vulgaris were observed in Western Europe (2583.9), high‐income Asia‐Pacific (2220.3), and Eastern Sub‐Saharan Africa (2188.7), while the lowest rates were found in Central Europe (838.8), Central Asia (959.5), and Eastern Europe (983.7). In 2021, the highest age‐standardized DALY rates (per 100 000 population) were observed in Western Europe (109.7), high‐income Asia‐Pacific (92.3), and Eastern Sub‐Saharan Africa (87.2), while the lowest rates were recorded in Central Europe (33.6), Central Asia (39.2), and Eastern Europe (40.2). From 1990 to 2021, the largest increases in age‐standardized prevalence of acne vulgaris lesions occurred in North Africa and the Middle East (20.6%), South Asia (18.8%), and East Asia (18%). Age‐standardized incidence rates for acne vulgaris increased across all regions, with the largest increases occurring in North Africa and the Middle East (21.6%), South Asia (18.9%), and East Asia (17.3%). From 1990 to 2021, age‐standardized DALY rates increased in all regions, with the largest increases occurring in North Africa and the Middle East (20.6%), South Asia (19.3%), and East Asia (18.3%) (Table 1).

### 3.3 Country and Regional Levels

**FIGURE 1:**
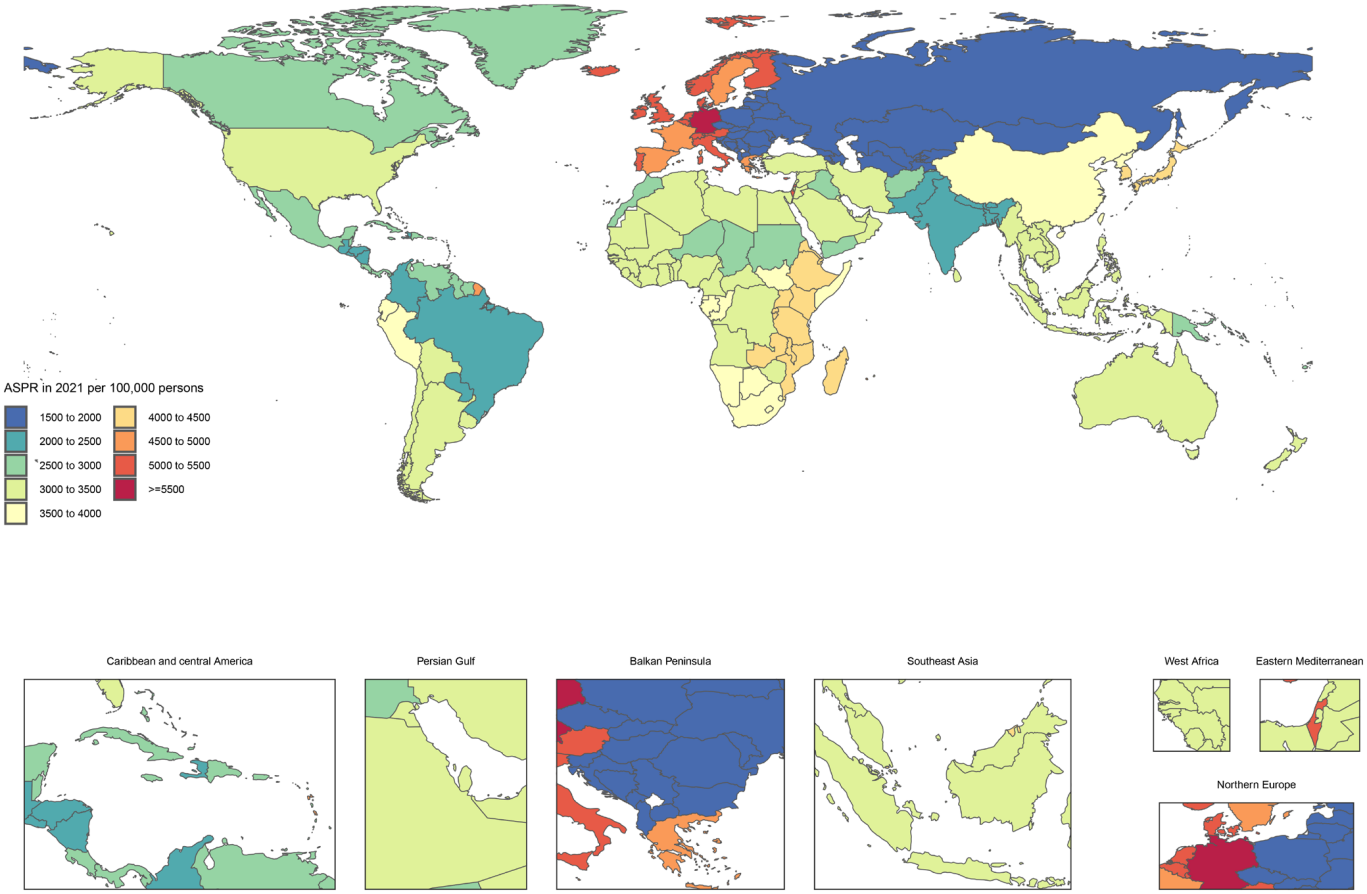
Age‐standardized point prevalence of acne vulgaris per 100 000 population by country, 2021 (based on https://ghdx.healthdata.org/gbd‐results‐tool).

**FIGURE 2:**
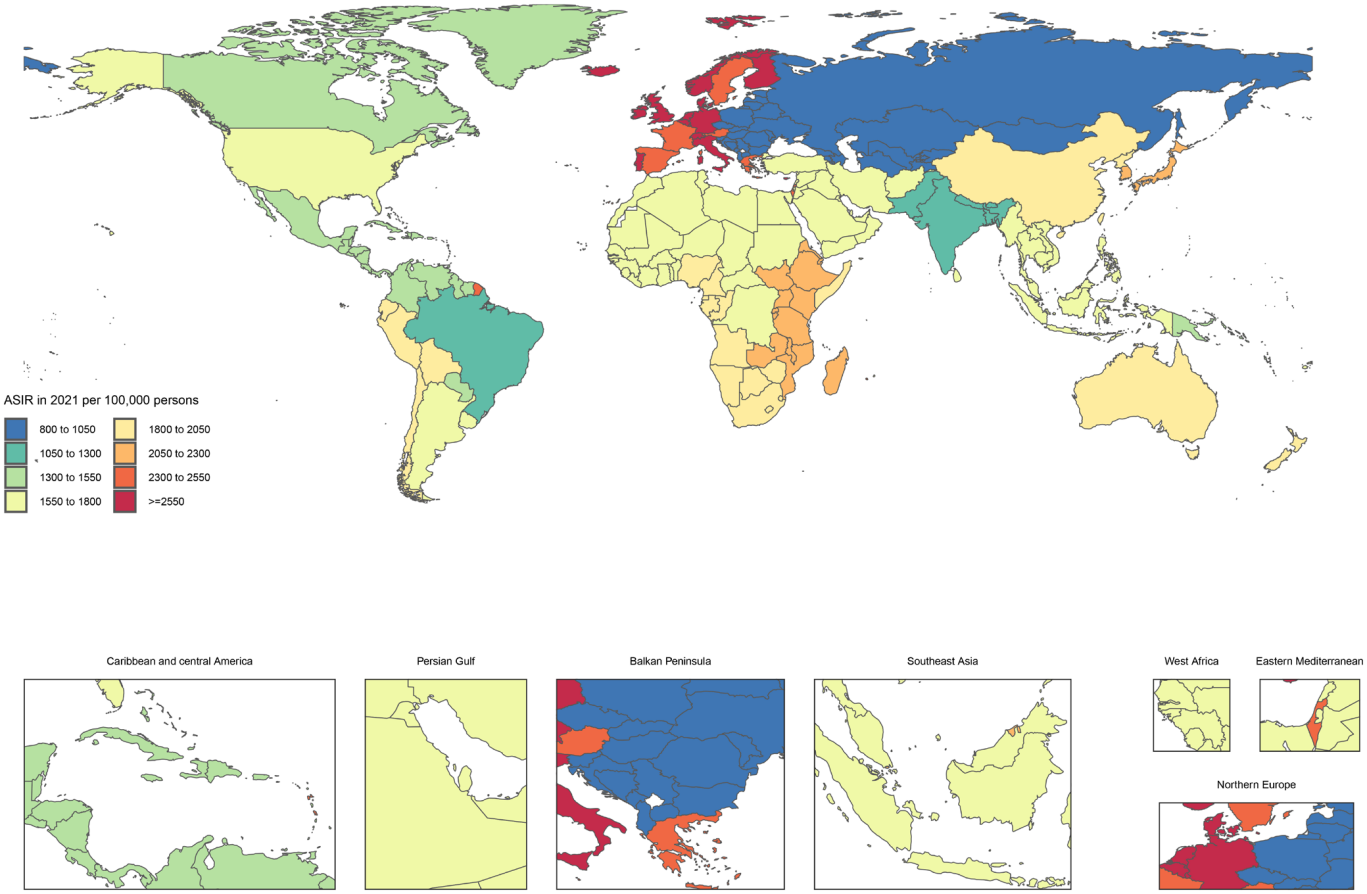
Age‐standardized incidence rate of acne vulgaris per 100 000 population in 2021, by country (based on https://ghdx.healthdata.org/gbd‐results‐tool).

In 2021, national age‐standardized prevalence rates for acne vulgaris ranged from 1500.3 to 5688.4 cases per 100 000 population. The highest age‐standardized prevalence rates were observed in Germany (5688.4), Portugal (5475.1), and Luxembourg (5323.1). Romania (1500.3), Albania (1519.6), and North Macedonia (1528.9) had the lowest age‐standardized prevalence rates (Table 2). In 2021, the national age‐standardized incidence rate of acne vulgaris ranged from 821.7 to 2723.0 cases per 100 000 population. Germany (2723.0), Portugal (2667.1), and Norway (2655.6) had the highest incidence rates, while Romania (821.7), Albania (826.6), and North Macedonia (830.6) had the lowest (Table 3). In 2021, the national age‐standardized DALY rate for acne vulgaris ranged from 32.2 to 121.0 per 100 000 population. Germany (121.0), Portugal (116.3), and Luxembourg (113.5) had the highest DALY rates, while Romania (32.2), Albania (32.6), and North Macedonia (32.9) had the lowest (Table 4). From 1990 to 2021, percentage changes in age‐standardized point prevalence rates varied significantly across countries, with Sudan (25.3%), Equatorial Guinea (25.2%), and Iraq (24.9%) experiencing the largest increases (Table 2). During the same period, the largest increases in age‐standardized incidence rates were observed in Sudan (25.0%), Iraq (25.0%), and Oman (24.5%) (Table 3). From 1990 to 2021, the largest increases in age‐standardized DALY rates for acne vulgaris were observed in Equatorial Guinea (26.5%), Sudan (25.4%), and Iraq (25.0%) (Table 4, Figures 1 and 2).

**TABLE 2:** Prevalent cases of acne vulgaris in 1990 and 2021 and the percentage change in the age‐standardised rates (ASRs) per 100,000, by location (generated from data available from http://ghdx.healthdata.org/gbd‐results‐tool).

Location_name	1990_No (95% UI)	1990_ASRs_per_100 000 (95% UI)	2021_No (95% UI)	2021_ASRs_per_100 000 (95% UI)	Percentage_change_in_the_ASRs_per_100 000
Bolivia (Plurinational State of)	236 782 (207 075, 271 637)	3005.3 (2646.4, 3432.5)	430 678 (383 178, 488 496)	3399 (3025.2, 3854.5)	13.1 (6.4, 19.5)
Ecuador	400 132 (353 675, 458 167)	3147 (2796.3, 3585.9)	667 342 (595 971, 752 650)	3533.8 (3153.6, 3985.4)	12.3 (6.6, 18.3)
Peru	934 686 (838 797, 1 050 889)	3407.3 (3071.5, 3813.1)	1 262 773 (1 135 629, 1 417 868)	3513.1 (3149.6, 3956.3)	3.1 (−3.1, 8.8)
Australia	523 850 (471 681, 586 536)	3159.1 (2824.8, 3565.3)	721 323 (656 551, 804 357)	3444.8 (3094.2, 3902.5)	9 (4.3, 13.9)
New Zealand	116 294 (106 168, 127 928)	3368.1 (3072.6, 3711.3)	148 714 (136 292, 162 181)	3404.9 (3091.9, 3756.4)	1.1 (−3.2, 5.6)
Antigua and Barbuda	1639 (1437, 1902)	2437.1 (2138.1, 2825.4)	2167 (1903, 2498)	2754.5 (2400.1, 3192.7)	13 (7.5, 19)
Bahamas	7895 (6880, 9208)	2537.2 (2217.4, 2950)	11 229 (9817, 12 896)	2819.9 (2472.2, 3238.8)	11.1 (5.6, 17.4)
Barbados	6440 (5662, 7450)	2438.3 (2140.3, 2825.3)	6155 (5371, 7119)	2636.8 (2278.5, 3073.5)	8.1 (2.7, 14.7)
Belize	5408 (4709, 6301)	2238.5 (1966.6, 2586.5)	13 026 (11 392, 15 078)	2538 (2230.9, 2932.6)	13.4 (7.6, 19.4)
Bermuda	1295 (1150, 1479)	2554.2 (2237.7, 2952.5)	1128 (1008, 1285)	2824.7 (2498.8, 3252.7)	10.6 (5.1, 17)
Cuba	275 278 (240 469, 315 480)	2303.2 (2015.9, 2628.9)	202 396 (180 127, 230 782)	2529 (2224.6, 2925.3)	9.8 (4.2, 16.1)
Dominica	2074 (1807, 2405)	2346 (2055, 2704.8)	1780 (1560, 2040)	2689.6 (2360.3, 3081.2)	14.6 (8.2, 21.3)
Dominican Republic	204 216 (177 351, 238 432)	2245.8 (1961.6, 2605.6)	286 394 (250 541, 328 743)	2555 (2234.3, 2938.9)	13.8 (7.6, 21.1)
Grenada	2333 (2017, 2712)	2272.4 (1976.6, 2626)	2585 (2279, 2966)	2632 (2314.7, 3033.5)	15.8 (9.7, 22)
Guyana	22 616 (19 742, 26 340)	2277.5 (1999.8, 2635.9)	20 834 (18 252, 24 104)	2604.8 (2285.3, 3020.7)	14.4 (7.8, 19.9)
Haiti	167 800 (146 221, 196 544)	2210.3 (1945.7, 2570.4)	365 016 (315 929, 421 928)	2477.4 (2149.9, 2856.9)	12.1 (6.2, 19.4)
Jamaica	68 065 (58 948, 79 107)	2321 (2022.4, 2680)	71 626 (62 861, 82 357)	2596.4 (2270.2, 2996.5)	11.9 (5.3, 18.8)
Puerto Rico	96 133 (84 505, 110 893)	2463.7 (2169.9, 2833.2)	68 658 (60 378, 79 082)	2755.1 (2406.2, 3191.1)	11.8 (6.3, 17.1)
Saint Kitts and Nevis	1180 (1029, 1364)	2396.6 (2100.9, 2756.3)	1346 (1187, 1542)	2714.4 (2375.1, 3135.6)	13.3 (7.4, 18.8)
Saint Lucia	4022 (3471, 4654)	2311.4 (2011.5, 2659.9)	3853 (3386, 4418)	2614.3 (2272.4, 3033)	13.1 (7.4, 19.1)
Saint Vincent and the Grenadines	3264 (2832, 3817)	2261.4 (1976.3, 2622.7)	2704 (2386, 3106)	2556.9 (2251.4, 2949.2)	13.1 (6.6, 18.8)
Suriname	10 744 (9362, 12 439)	2312.4 (2025.7, 2667.9)	14 463 (12 654, 16 729)	2563.8 (2243.2, 2969.6)	10.9 (4.8, 16.8)
Trinidad and Tobago	32 744 (28 708, 37 809)	2409 (2116.1, 2775.7)	30 847 (27 456, 35 253)	2683.3 (2373.4, 3077.3)	11.4 (6.8, 17)
United States Virgin Islands	2841 (2490, 3274)	2490.3 (2188.1, 2861.6)	1651 (1470, 1891)	2765.2 (2441.8, 3185.6)	11 (5.6, 16.7)
Armenia	57 290 (50 178, 65 524)	1632 (1423.8, 1872.4)	41 521 (36 764, 47 227)	1803 (1577.2, 2067.8)	10.5 (5.3, 16.3)
Azerbaijan	141 959 (124 054, 163 444)	1702.2 (1489, 1962.4)	169 379 (150 244, 191 867)	1836.8 (1612.7, 2094.5)	7.9 (2.3, 13.2)
Georgia	90 579 (79 430, 103 950)	1727.5 (1508.7, 1989.6)	47 104 (41 661, 53 587)	1807.5 (1586.8, 2072.4)	4.6 (−0.4, 11.4)
Kazakhstan	296 260 (257 171, 341 085)	1694.8 (1470.9, 1949.6)	311 566 (272 722, 354 094)	1849.4 (1616.1, 2101.1)	9.1 (3.9, 15.2)
Kyrgyzstan	84 553 (72 876, 97 503)	1667.5 (1444, 1914)	124 916 (109 439, 144 294)	1783.4 (1560.3, 2057.7)	7 (1.6, 12.4)
Mongolia	43 803 (38 202, 51 528)	1615.5 (1413.9, 1883.5)	54 893 (48 348, 63 051)	1801.7 (1582.9, 2077.8)	11.5 (5.4, 17.4)
Tajikistan	104 166 (90 880, 121 485)	1632.4 (1433, 1888.9)	190 549 (165 633, 219 570)	1752 (1523.1, 2018.3)	7.3 (1.2, 14)
Turkmenistan	74 873 (65 648, 85 865)	1684.8 (1486.6, 1921.4)	98 207 (86 145, 112 985)	1844.6 (1616.5, 2124.9)	9.5 (3.6, 16.1)
Uzbekistan	407 392 (355 271, 472 776)	1647.2 (1444.4, 1903)	596 029 (518 914, 678 221)	1836.8 (1592.4, 2099.9)	11.5 (5.6, 17.6)
Albania	52 225 (47 707, 57 629)	1355.1 (1239.1, 1494.5)	33 084 (30 416, 36 415)	1519.6 (1382.4, 1689.7)	12.1 (7.2, 17.2)
Bosnia and Herzegovina	61 883 (56 662, 68 309)	1332.3 (1216.1, 1475.3)	35 535 (32 616, 38 817)	1553.4 (1410.8, 1710.7)	16.6 (11.3, 22.3)
Bulgaria	109 616 (99 821, 120 106)	1446.2 (1313.7, 1589.3)	69 439 (64 023, 75 301)	1641.1 (1503.2, 1789.5)	13.5 (7.5, 20)
Croatia	58 679 (53 477, 64 486)	1377.5 (1249.1, 1522.5)	43 019 (39 532, 47 054)	1540.3 (1405.5, 1700.7)	11.8 (5.9, 17.4)
Czechia	135 785 (124 043, 149 192)	1386.8 (1267.3, 1521.5)	106 100 (96 812, 116 346)	1567.4 (1423.6, 1729.6)	13 (8, 19.4)
Hungary	128 586 (117 805, 142 276)	1370.9 (1254.6, 1516.4)	97 989 (89 908, 106 797)	1553.1 (1415, 1702.9)	13.3 (8.1, 18.5)
Montenegro	8956 (8169, 9904)	1405.4 (1280.9, 1555.7)	7711 (7082, 8483)	1578.8 (1447.7, 1744.1)	12.3 (7.2, 17.8)
North Macedonia	27 888 (25 467, 30 835)	1362.2 (1242.8, 1506.7)	24 288 (22 449, 26 654)	1528.9 (1401.3, 1696.3)	12.2 (6.4, 17.8)
Poland	539 726 (502 064, 583 886)	1530.2 (1422.8, 1656.3)	403 644 (389 234, 420 596)	1596.8 (1531.5, 1672.1)	4.4 (−0.6, 9.6)
Romania	303 203 (275 804, 339 429)	1308.8 (1191.4, 1464.4)	196 424 (180 577, 216 783)	1500.3 (1373.7, 1663.2)	14.6 (9.4, 20.2)
Serbia	123 691 (112 808, 135 605)	1384.6 (1260, 1521.4)	106 824 (97 896, 117 518)	1558.9 (1417.1, 1725.2)	12.6 (7.6, 17.9)
Slovakia	71 648 (65 571, 79 160)	1382.9 (1263.7, 1525.9)	56 543 (52 091, 61 421)	1560.1 (1425.5, 1712.5)	12.8 (7.7, 18.2)
Slovenia	26 317 (24 021, 28 554)	1455.1 (1320.3, 1586.9)	20 477 (18 787, 22 385)	1610.4 (1465.7, 1770.8)	10.7 (5.3, 17.1)
Colombia	859 922 (745 754, 1 003 597)	2182.1 (1900.4, 2536.5)	1 156 305 (1 019 341, 1 328 578)	2472.9 (2163.4, 2853.3)	13.3 (5.6, 20.5)
Costa Rica	80 179 (70 349, 92 812)	2253.6 (1981.4, 2600.4)	110 837 (98 110, 126 196)	2543 (2233.3, 2912.9)	12.8 (7.2, 18.5)
El Salvador	150 485 (130 514, 176 115)	2190.6 (1923.5, 2539.6)	168 114 (147 284, 192 591)	2490.3 (2180.6, 2853.7)	13.7 (7.9, 19.6)
Guatemala	221 009 (189 848, 257 051)	2107.5 (1829, 2427.2)	458 248 (400 243, 533 708)	2417.9 (2119.2, 2806)	14.7 (8.8, 20.9)
Honduras	129 577 (111 975, 152 137)	2150.8 (1883.9, 2496.1)	296 848 (258 136, 342 824)	2425.3 (2119.8, 2788.5)	12.8 (7.1, 18.7)
Mexico	2 554 452 (2 284 360, 2 905 246)	2281.5 (2052.8, 2575.5)	3 335 605 (3 014 705, 3 754 013)	2537 (2293.8, 2855.2)	11.2 (9.8, 12.6)
Nicaragua	108 507 (93 678, 128 389)	2132.4 (1857.7, 2496.7)	179 060 (157 606, 205 836)	2432.1 (2140.9, 2794.8)	14.1 (7.8, 21)
Panama	68 973 (60 293, 80 733)	2369.5 (2080.9, 2758.8)	115 388 (101 178, 133 552)	2679.4 (2345.3, 3105.1)	13.1 (7.6, 19.5)
Venezuela (Bolivarian Republic of)	537 949 (470 951, 628 664)	2316.8 (2037.2, 2693.7)	637 465 (559 856, 742 090)	2547 (2240.2, 2956)	9.9 (4, 15.8)
Angola	364 141 (318 125, 420 181)	2953.3 (2609.2, 3383.2)	1 421 805 (1 241 052, 1 645 045)	3490.1 (3074.9, 4006.8)	18.2 (13, 23.5)
Central African Republic	94 436 (82 517, 108 649)	2930 (2578.3, 3355.5)	226 612 (196 176, 264 053)	3256.7 (2855.4, 3756.5)	11.1 (6.1, 17.5)
Congo	97 314 (85 004, 113 218)	3146.6 (2776.7, 3631)	235 804 (206 894, 274 082)	3617.1 (3198.1, 4184.8)	15 (9.2, 20.6)
Democratic Republic of the Congo	1 371 615 (1 195 834, 1 590 069)	2989.8 (2625, 3432.4)	3 807 218 (3 323 883, 4 418 824)	3325.3 (2925, 3825.7)	11.2 (4.9, 18.8)
Equatorial Guinea	14 693 (12 850, 17 080)	2943.8 (2599.1, 3384.5)	76 448 (66 745, 88 336)	3684.7 (3248.6, 4223.1)	25.2 (17.6, 32.9)
Gabon	36 580 (31 729, 42 359)	3116.6 (2727.7, 3582.8)	80 931 (70 891, 93 071)	3712.9 (3270.9, 4244.6)	19.1 (12.2, 26)
China	42 619 853 (38 256 909, 47 529 058)	3166.6 (2844.6, 3522.4)	39 458 445 (36 080 238, 43 300 754)	3742.2 (3380.3, 4152.5)	18.2 (16.3, 19.9)
Democratic People's Republic of Korea	673 538 (601 756, 763 974)	3167.1 (2827, 3601.9)	797 638 (711 136, 889 387)	3530.7 (3116.1, 3968.2)	11.5 (6.3, 16.8)
Belarus	154 842 (135 847, 176 899)	1682.6 (1469.1, 1932.7)	115 747 (102 579, 131 393)	1868.6 (1628.7, 2142.4)	11.1 (4.9, 17.5)
Estonia	23 091 (20 181, 26 640)	1699.4 (1478.5, 1969.9)	16 478 (14 408, 18 600)	1892.2 (1639.6, 2151.8)	11.3 (5.9, 17.5)
Latvia	38 470 (33 834, 44 019)	1705.8 (1490.7, 1969.5)	22 665 (19 983, 25 607)	1899.2 (1655.3, 2163)	11.3 (5.7, 16.6)
Lithuania	57 674 (50 479, 65 831)	1710.5 (1486.2, 1963.9)	33 950 (30 196, 38 438)	1923.2 (1691.9, 2206.8)	12.4 (6.9, 19)
Republic of Moldova	70 641 (61 638, 81 496)	1667.8 (1452.9, 1926.3)	44 778 (39 552, 50 748)	1844 (1604.5, 2116.1)	10.6 (5.4, 16)
Russian Federation	2 281 601 (2 064 691, 2 558 890)	1727.2 (1553.9, 1948.8)	1 923 432 (1 750 154, 2 152 894)	1885.2 (1700.1, 2123.9)	9.1 (8.1, 10.3)
Ukraine	767 883 (685 641, 871 917)	1697.6 (1511.5, 1940.4)	531 413 (478 180, 594 580)	1838.7 (1636.2, 2081.6)	8.3 (3.8, 13.4)
Burundi	234 241 (207 823, 267 145)	3622.3 (3235.2, 4098.1)	668 775 (589 410, 763 256)	4001 (3561.5, 4519.4)	10.5 (4.7, 17.1)
Comoros	21 816 (19 273, 25 006)	3709.9 (3310.2, 4206.6)	36 985 (32 983, 41 776)	4306.6 (3852.1, 4848.6)	16.1 (10.5, 21.8)
Djibouti	19 769 (17 514, 22 613)	3550.4 (3168.5, 4016.6)	57 193 (51 175, 64 759)	4080.3 (3652.5, 4617.7)	14.9 (9.6, 20.5)
Eritrea	154 964 (136 930, 178 153)	3589.6 (3214.1, 4085.3)	335 314 (298 486, 379 348)	4168.4 (3728.9, 4697.7)	16.1 (10.9, 21.6)
Ethiopia	2 144 344 (1 942 270, 2 401 609)	3480.5 (3188.3, 3871)	5 823 360 (5 264 688, 6 475 036)	4046.1 (3678.8, 4467.8)	16.2 (13.1, 19)
Kenya	1 091 746 (982 429, 1 220 617)	3621.5 (3296.6, 4015.1)	2 764 406 (2 507 397, 3 072 043)	4161.9 (3806.7, 4587.7)	14.9 (13.6, 16.3)
Madagascar	537 854 (474 250, 614 689)	3653.5 (3252.5, 4134.6)	1 515 509 (1 346 396, 1 727 268)	4115.1 (3676.3, 4657)	12.6 (7.7, 18.4)
Malawi	428 963 (378 772, 487 094)	3585.1 (3198.9, 4045.1)	1 118 395 (995 152, 1 268 291)	4117.7 (3701, 4629.9)	14.9 (10.2, 20.2)
Mozambique	562 642 (497 651, 645 250)	3489.2 (3115.3, 3972.7)	1 627 151 (1 447 472, 1 847 763)	4027.7 (3612.2, 4529.8)	15.4 (10.2, 21.1)
Rwanda	325 310 (290 064, 370 719)	3725.3 (3349.3, 4202.4)	706 794 (627 629, 793 426)	4250.5 (3796.6, 4746.1)	14.1 (8.7, 19.5)
Somalia	327 992 (289 519, 377 554)	3286.1 (2936.6, 3744.3)	985 058 (869 786, 1 111 396)	3532.4 (3147.8, 3956.8)	7.5 (1.8, 14.6)
South Sudan	268 157 (238 384, 306 228)	3556.5 (3189.5, 4026)	512 207 (450 670, 587 810)	3911.9 (3486.2, 4443.4)	10 (4.3, 16.7)
Uganda	750 356 (658 583, 858 943)	3487.4 (3095.1, 3957.4)	2 381 564 (2 131 476, 2 707 703)	4122.1 (3722.1, 4628.9)	18.2 (12.7, 23.9)
United Republic of Tanzania	1 230 296 (1 093 767, 1 399 295)	3776.5 (3385.4, 4259.6)	3 097 640 (2 756 419, 3 490 083)	4186.4 (3760.3, 4676.1)	10.9 (5.3, 17.3)
Zambia	388 821 (343 395, 441 903)	3701.8 (3315.5, 4167.6)	1 089 004 (963 630, 1 243 349)	4315.3 (3852.3, 4880.6)	16.6 (10.6, 23.1)
Brunei Darussalam	11 312 (10 096, 12 685)	3821.8 (3406.7, 4278.4)	19 086 (17 492, 21 110)	4221.7 (3832, 4728)	10.5 (4.9, 15.4)
Japan	4 763 216 (4 398 810, 5 234 128)	4020.5 (3694, 4431.3)	3 597 664 (3 361 687, 3 885 815)	4339.6 (3991, 4761)	7.9 (6.5, 9.5)
Republic of Korea	2 006 022 (1 781 958, 2 252 313)	3884 (3457.2, 4376.3)	1 568 335 (1 444 576, 1 708 798)	4237.7 (3826.7, 4726.1)	9.1 (4.6, 14.5)
Singapore	129 156 (115 565, 144 013)	3864.2 (3449, 4344.9)	171 138 (158 372, 187 543)	4271.7 (3843.6, 4813.3)	10.5 (5.7, 15.1)
Canada	689 659 (644 421, 747 677)	2645.1 (2450.9, 2892.8)	855 229 (800 620, 920 358)	2863.3 (2650, 3128.6)	8.2 (4, 12.9)
Greenland	1437 (1344, 1555)	2519.1 (2329.9, 2770.5)	1389 (1299, 1500)	2789.5 (2579.9, 3045)	10.7 (6.3, 15.4)
United States of America	6 968 940 (6 594 272, 7 446 620)	2873.4 (2705.3, 3099.5)	8 970 041 (8 649 545, 9 363 273)	3128.5 (3004.2, 3277.4)	8.9 (4.8, 13.5)
Afghanistan	309 993 (270 380, 359 287)	2390.3 (2124.8, 2722.3)	1 154 483 (1 007 399, 1 330 841)	2843 (2504.9, 3251.7)	18.9 (13.2, 25.8)
Algeria	840 196 (740 033, 956 867)	2608.5 (2317.2, 2946.4)	1 299 964 (1 162 828, 1 471 705)	3128.9 (2788.3, 3557.6)	19.9 (14, 26.2)
Bahrain	13 956 (12 538, 15 737)	2693.5 (2392.9, 3056.1)	44 665 (40 427, 50 030)	3209 (2867.5, 3638.2)	19.1 (13.6, 25.8)
Egypt	1 844 564 (1 626 099, 2 107 159)	2778.3 (2466.7, 3159)	3 795 284 (3 357 591, 4 332 645)	3252.4 (2882.1, 3699.5)	17.1 (11.3, 22.9)
Iran (Islamic Republic of)	1 815 170 (1 644 488, 2 036 529)	2562.6 (2335.4, 2852.8)	2 369 321 (2 171 295, 2 620 476)	3143.9 (2859, 3497.3)	22.7 (21, 24.3)
Iraq	538 576 (469 356, 618 006)	2296.5 (2021.1, 2621.2)	1 402 658 (1 225 433, 1 589 852)	2868.5 (2513.8, 3241.1)	24.9 (18.6, 32.3)
Jordan	133 870 (118 217, 153 524)	2635.2 (2350.1, 2998.7)	462 298 (410 730, 530 227)	3124.7 (2783, 3572.5)	18.6 (12.9, 25.2)
Kuwait	50 262 (44 742, 57 058)	2775.7 (2447.7, 3175.4)	123 399 (111 198, 138 036)	3311.7 (2933.3, 3789.2)	19.3 (12.8, 26.5)
Lebanon	86 861 (77 116, 98 651)	2573.7 (2297.2, 2908.1)	157 392 (140 856, 176 628)	3129.5 (2772, 3531.3)	21.6 (16, 27.8)
Libya	143 780 (126 396, 164 562)	2562 (2280.7, 2905.6)	224 401 (200 367, 252 631)	3170.4 (2822.2, 3583.8)	23.7 (17.8, 30.3)
Morocco	773 746 (684 791, 879 225)	2489.9 (2221.3, 2813.1)	1 107 773 (978 071, 1 261 058)	2959.2 (2608.2, 3373.2)	18.8 (13.1, 25.1)
Oman	56 438 (50 318, 63 782)	2618.6 (2338, 2956.9)	133 559 (120 534, 148 674)	3265 (2891.6, 3696.4)	24.7 (18.8, 30.7)
Palestine	64 023 (56 811, 73 405)	2490.2 (2225.8, 2834.1)	193 323 (172 122, 220 805)	3036.9 (2715.7, 3449.7)	22 (16.1, 28)
Qatar	11 379 (10 246, 12 723)	2735.2 (2428, 3099.6)	69 189 (63 481, 76 408)	3282.9 (2923.2, 3775.9)	20 (14.8, 26.2)
Saudi Arabia	524 276 (461 911, 604 947)	2702.6 (2397.4, 3103.6)	1 160 126 (1 044 877, 1 316 833)	3349.8 (2976.5, 3865.4)	23.9 (18, 30.8)
Sudan	592 939 (522 080, 679 968)	2391.2 (2122.6, 2725.5)	1 655 983 (1 451 569, 1 886 548)	2995.2 (2639.8, 3387.6)	25.3 (18.3, 32.2)
Syrian Arab Republic	409 715 (361 311, 467 490)	2483.1 (2211.3, 2811.8)	545 184 (480 066, 623 480)	3047.3 (2711.1, 3452)	22.7 (15.8, 29.3)
Tunisia	264 242 (234 922, 301 221)	2582 (2307, 2925.3)	325 312 (291 655, 368 504)	3120.2 (2781.5, 3548.7)	20.8 (15, 27)
United Arab Emirates	48 169 (43 377, 54 337)	2755.6 (2450.7, 3147.1)	197 633 (179 715, 219 913)	3362.5 (2970.8, 3854)	22 (16.6, 27.2)
Yemen	383 602 (336 502, 442 540)	2340.4 (2072.5, 2664.6)	1 247 756 (1 098 429, 1 427 574)	2896.7 (2571.2, 3288.1)	23.8 (17.6, 30.1)
American Samoa	1570 (1367, 1792)	2757.4 (2415.9, 3137.5)	1688 (1481, 1961)	3035.2 (2688.6, 3487.3)	10.1 (5.4, 14.9)
Cook Islands	621 (543, 718)	2742.3 (2412.7, 3146.7)	504 (443, 574)	3088.9 (2726.2, 3516.4)	12.6 (8.2, 17.3)
Fiji	24 119 (21 006, 27 587)	2630.2 (2302.1, 3001.5)	28 307 (25 034, 32 374)	2966.7 (2622.9, 3392)	12.8 (7.4, 17.6)
Guam	4182 (3699, 4773)	2797.2 (2474.9, 3209.2)	4486 (3971, 5093)	3118 (2751.8, 3551.1)	11.5 (6.4, 15.9)
Kiribati	2102 (1837, 2412)	2478.7 (2172.4, 2836.9)	3764 (3275, 4294)	2720.5 (2372.8, 3099)	9.8 (5, 16.1)
Marshall Islands	1495 (1298, 1728)	2547.7 (2233.6, 2913.2)	1845 (1619, 2125)	2850.6 (2509.7, 3269.9)	11.9 (7.3, 17.1)
Micronesia (Federated States of)	3459 (3029, 3992)	2594.6 (2295.5, 2966.7)	3470 (3047, 3980)	2878.6 (2543.5, 3277.8)	10.9 (6.4, 15.1)
Nauru	310 (272, 352)	2640.5 (2321.3, 2982.2)	387 (338, 441)	2930.9 (2575, 3329.1)	11 (6.1, 16)
Niue	67 (59, 77)	2678.3 (2344.1, 3048.9)	48 (43, 54)	3010.5 (2666.6, 3393.6)	12.4 (7.6, 17.6)
Northern Mariana Islands	1425 (1261, 1627)	2887.6 (2548, 3317.8)	1424 (1264, 1618)	3089.9 (2741.9, 3509.5)	7 (2.8, 12.7)
Palau	501 (442, 572)	2770.4 (2452.6, 3160)	453 (400, 518)	3052.2 (2672.3, 3506.9)	10.2 (5.3, 15.2)
Papua New Guinea	120 324 (105 186, 139 141)	2411.9 (2124.5, 2762.6)	315 302 (275 957, 360 755)	2669.1 (2342.3, 3047.3)	10.7 (6.4, 15.9)
Samoa	5864 (5102, 6783)	2637.8 (2326.9, 3016.1)	7081 (6205, 8101)	2908 (2561.1, 3304.4)	10.2 (5.1, 14.9)
Solomon Islands	10 528 (9103, 12 169)	2413 (2114, 2764.9)	21 899 (19 023, 25 436)	2693.5 (2354.2, 3101.9)	11.6 (7.1, 16.8)
Tokelau	46 (40, 53)	2564.5 (2244.4, 2946.1)	43 (38, 49)	2920.8 (2577.4, 3324.5)	13.9 (8.5, 19.1)
Tonga	3327 (2897, 3842)	2635.3 (2323.3, 3016.2)	3535 (3064, 4054)	2945 (2570.2, 3350.3)	11.8 (6.6, 17.7)
Tuvalu	246 (216, 282)	2556.3 (2238.6, 2918.7)	388 (343, 444)	2869.6 (2541, 3266.3)	12.3 (7.9, 17.1)
Vanuatu	4517 (3925, 5187)	2484.1 (2176, 2832.5)	10 034 (8823, 11 488)	2771.9 (2444.9, 3156.3)	11.6 (6, 16.9)
Bangladesh	2 547 391 (2 233 539, 2 924 736)	1928.8 (1710.2, 2192.8)	4 122 112 (3 626 491, 4 748 226)	2285.5 (2012.5, 2631.9)	18.5 (12.9, 23.9)
Bhutan	15 599 (13 527, 17 968)	1829.4 (1611.2, 2081.8)	17 682 (15 621, 20 074)	2174.1 (1915.2, 2476.6)	18.8 (12.6, 25.1)
India	19 764 304 (17 818 421, 22 304 167)	1998.3 (1808.4, 2245.5)	37 433 701 (33 758 639, 42 025 930)	2388.2 (2155.8, 2683.5)	19.5 (18.2, 20.9)
Nepal	404 518 (351 044, 469 373)	1776.5 (1551.5, 2046.8)	751 043 (649 549, 869 686)	2062.5 (1792.1, 2381.1)	16.1 (10.7, 22.8)
Pakistan	2 402 727 (2 152 849, 2 716 623)	1778.3 (1603.7, 1992.4)	5 864 505 (5 288 191, 6 604 106)	2094.9 (1897, 2350.4)	17.8 (14.2, 21.3)
Cambodia	334 148 (291 659, 380 797)	2737 (2420.2, 3095.9)	567 886 (501 819, 643 372)	3095.8 (2735.9 3507.8)	13.1 (8.7, 18.3)
Indonesia	6 682 662 (6 006 557, 7 516 402)	2944.3 (2663.1, 3293.1)	9 393 080 (8 525 146, 10 462 176)	3383.8 (3061.3, 3780)	14.9 (13.4, 16.6)
Lao People's Democratic Republic	133 356 (116 899, 152 862)	2689.9 (2388, 3051.6)	251 094 (221 206, 286 148)	3079.4 (2717, 3505)	14.5 (9.5, 19.8)
Malaysia	611 062 (540 480, 697 809)	2951 (2623.3, 3356.2)	1 081 532 (966 456, 1 213 580)	3351.6 (2986.4, 3792.8)	13.6 (8.7, 18.3)
Maldives	7179 (6300, 8225)	2664.8 (2357.8, 3033.8)	14 226 (12 857, 15 839)	3143.1 (2792.6, 3548.7)	17.9 (12.6, 23.7)
Mauritius	36 549 (32 449, 41 501)	2913.5 (2592.2, 3306.9)	35 966 (32 224, 40 498)	3363.8 (2992.4, 3814.4)	15.5 (9.8, 20.8)
Myanmar	1 358 510 (1 200 385, 1 556 112)	2744.8 (2441.1, 3118.6)	1 898 421 (1 682 705, 2 167 620)	3180.7 (2823.1, 3623.8)	15.9 (10.8, 20.9)
Philippines	2 266 434 (2 041 134, 2 544 672)	2923.2 (2649.1, 3261.7)	4 114 242 (3 725 157, 4 607 376)	3259.6 (2957.9, 3641.6)	11.5 (10.5, 12.9)
Seychelles	2606 (2311, 2986)	3053.2 (2721.9, 3484.5)	3115 (2801, 3517)	3382.4 (3015.4, 3844.3)	10.8 (6.7, 15.8)
Sri Lanka	577 971 (512 815, 664 060)	2886.4 (2570.4, 3302.6)	685 933 (606 849, 786 508)	3236.1 (2861.9, 3712.5)	12.1 (7.9, 16.7)
Thailand	1 890 657 (1 677 197, 2 162 015)	2782.1 (2478.7, 3170.1)	1 524 474 (1 353 511, 1 706 358)	3051.8 (2677.5, 3451.5)	9.7 (5.3, 15.2)
Timor‐Leste	23 976 (21 154, 27 290)	2719.6 (2407.7, 3088.9)	56 778 (50 131, 65 195)	3220.8 (2864.1, 3663.6)	18.4 (12.7, 23.8)
Viet Nam	2 343 448 (2 064 399, 2 683 331)	2850.9 (2522.6, 3242.8)	2 955 607 (2 650 061, 3 367 648)	3263.7 (2911, 3742.5)	14.5 (10.1, 19.2)
Uruguay	98 089 (87 723, 110 159)	3197.4 (2860.1, 3589.9)	105 453 (95 067, 117 366)	3462.2 (3101.8, 3893)	8.3 (3.7, 13.5)
Argentina	1 078 380 (968 069, 1 221 435)	3175.4 (2862.3, 3579.3)	1 529 405 (1 372 210, 1 710 101)	3460.6 (3079.5, 3895.2)	9 (4.3, 13.6)
Chile	467 108 (416 477, 530 042)	3188.9 (2849.3, 3608.6)	592 167 (535 423, 657 609)	3485.9 (3110.1, 3934.3)	9.3 (3.9, 14.1)
Botswana	53 909 (46 613, 62 195)	3134.7 (2750.5, 3582.7)	95 426 (84 692, 107 997)	3642.8 (3232.1, 4127.6)	16.2 (11.2, 21.9)
Eswatini	31 750 (27 621, 37 252)	3027.1 (2670.4, 3505.1)	50 117 (43 826, 57 945)	3523.9 (3098.5, 4055)	16.4 (10.8, 21.6)
Lesotho	56 229 (48 452, 64 836)	3030.5 (2647.7, 3465.3)	82 482 (72 092, 95 595)	3509.5 (3080.4, 4039.4)	15.8 (10.2, 21.8)
Namibia	56 539 (49 277, 65 442)	3136.6 (2761.8, 3599.5)	105 093 (92 412, 121 276)	3632.7 (3211.7, 4174)	15.8 (10.5, 21.3)
South Africa	1 471 597 (1 318 333, 1 662 686)	3241.7 (2918.8, 3644.7)	2 127 405 (1 938 118, 2 371 294)	3660.7 (3326.1, 4088.5)	12.9 (10.9, 15.4)
Zimbabwe	423 947 (367 615, 494 996)	3104.5 (2722.2, 3575.3)	672 711 (586 550, 777 838)	3431.1 (3008.9, 3946.7)	10.5 (5.2, 17.3)
Brazil	3 824 121 (3 440 624, 4 332 989)	2108.9 (1904.4, 2379.7)	4 521 731 (4 092 244, 5 069 623)	2335.5 (2101.1, 2630.9)	10.7 (9, 12.3)
Paraguay	106 140 (92 424, 123 490)	2196.5 (1923.8, 2549.8)	194 938 (170 205, 223 354)	2496.6 (2183.9, 2858.9)	13.7 (6.8, 20.2)
Andorra	2601 (2376, 2854)	4816.5 (4343.3, 5344.9)	3255 (3003, 3570)	5171 (4693.9, 5789.4)	7.4 (2.6, 12.6)
Austria	326 983 (300 127, 359 083)	4711.7 (4282.6, 5248.2)	334 496 (308 168, 362 769)	5048.9 (4587.6, 5553.6)	7.2 (2.8, 11.6)
Belgium	408 466 (373 185, 450 809)	4665.3 (4230.1, 5211.3)	445 565 (408 475, 488 766)	5071.2 (4592.6, 5638.9)	8.7 (3.4, 13.7)
Cyprus	35 515 (32 255, 39 598)	4599.5 (4161, 5143.2)	55 124 (51 120, 60 038)	5155 (4666.6, 5735.4)	12.1 (6.8, 17.2)
Denmark	224 832 (203 219, 248 631)	4821 (4327.7, 5389.8)	238 038 (218 734, 261 077)	5172.3 (4695.2, 5741.2)	7.3 (2.7, 12)
Finland	204 451 (188 349, 224 114)	4751.1 (4326.2, 5283.9)	213 769 (194 728, 234 868)	5143.6 (4626, 5733.7)	8.3 (4, 13.7)
France	2 367 310 (2 142 432, 2 639 997)	4424.2 (3992.3, 4958.2)	2 521 767 (2 282 466, 2 814 230)	4799.8 (4316.8, 5407.4)	8.5 (3.5, 13.6)
Germany	3 597 770 (3 312 372, 3 908 699)	5401.6 (4882.4, 5995.9)	3 412 445 (3 139 087, 3 709 033)	5688.4 (5144.8, 6297)	5.3 (1, 10.1)
Greece	426 350 (383 611, 478 616)	4413.1 (3948.4, 4977.4)	348 260 (318 068, 384 774)	4864.7 (4375.6, 5476.3)	10.2 (5.3, 16)
Iceland	11 989 (10 859,13 286)	4669 (4214.4, 5196)	15 079 (13 795, 16 645)	5104.8 (4636.7, 5693.3)	9.3 (4.6, 15)
Ireland	179 362 (161 772, 200 727)	4672 (4232.8, 5202.1)	216 859 (197 604, 238 928)	5162.5 (4675.6, 5730.5)	10.5 (5.8, 15.2)
Israel	247 567 (221 532, 277 837)	4672.4 (4202, 5213.6)	456 606 (415 637, 509 289)	5057.6 (4600.3, 5641.6)	8.2 (3.8, 12.8)
Italy	2 497 213 (2 311 400, 2 712 301)	4738.4 (4349, 5196.9)	2 065 248 (1 929 972, 2 216 722)	5059.8 (4658.4, 5505.4)	6.8 (5, 8.7)
Luxembourg	16 026 (14 728, 17 558)	4923.7 (4427.1, 5484.2)	26 929 (24 592, 29 418)	5323.1 (4789.3, 5931.2)	8.1 (3.6, 13.3)
Malta	15 754 (14 321, 17 463)	4497.9 (4067.3, 5009.2)	15 046 (13 969, 16 495)	4903 (4463.9, 5496.5)	9 (4.8, 13.6)
Monaco	1012 (937, 1090)	4833 (4385.4, 5364.3)	1270 (1170, 1393)	5148.6 (4673.4, 5736.2)	6.5 (2.3, 11.3)
Netherlands	665 480 (609 766, 731 163)	4745.6 (4302.9, 5278)	685 505 (630 481, 755 667)	5149.7 (4687.2, 5746.3)	8.5 (4.4, 13.3)
Norway	187 162 (173 049, 202 805)	4835.1 (4421.5, 5291.4)	230 082 (213 565, 248 993)	5268.6 (4837.7, 5757.9)	9 (7, 11)
Portugal	505 763 (457 884, 561 878)	5006.1 (4522.9, 5572.4)	417 465 (382 206, 454 466)	5475.1 (4942.6, 6060.3)	9.4 (5.3, 13.6)
San Marino	1117 (1013, 1233)	4843.6 (4364.2, 5415.9)	1260 (1151, 1386)	5207.1 (4708.6, 5789.9)	7.5 (3.2, 12.5)
Spain	1 788 107 (1 616 774, 2 003 693)	4581.3 (4132.9, 5150.5)	1 624 249 (1 496 398, 1 775 588)	4970 (4517.4, 5526.6)	8.5 (3.6, 13.9)
Sweden	318 246 (293 442, 345 442)	4442.4 (4074.2, 4866.1)	382 977 (355 712, 416 069)	4777.4 (4401.1, 5243.3)	7.5 (3.7, 11.9)
Switzerland	290 657 (266 621, 318 829)	4898.7 (4408.4, 5480.8)	333 862 (308 233, 365 284)	5154.8 (4679.2, 5755.9)	5.2 (0.8, 10.4)
United Kingdom	2 391 711 (2 217 457, 2 590 111)	4747.1 (4351.2, 5194.9)	2 777 090 (2 583 696, 3 005 451)	5173.6 (4765.1, 5654.2)	9 (7.6, 10.6)
Benin	153 727 (136 312, 176 623)	2763.1 (2467.2, 3144.8)	531 910 (468 016, 605 189)	3165.3 (2804, 3580.2)	14.6 (9, 20.6)
Burkina Faso	300 402 (262 718, 342 118)	2644.9 (2332.6, 2988)	861 281 (757 835, 980 676)	3058.7 (2717.6, 3456.9)	15.6 (10.7, 22.3)
Cabo Verde	12 027 (10 591, 13 756)	2783.7 (2471.8, 3159.2)	19 753 (17 562, 22 348)	3305.8 (2933.6, 3743.7)	18.8 (12.4, 24.8)
Cameroon	353 622 (308 980, 409 384)	2853.3 (2503.8, 3282.9)	1 356 424 (1 199 253, 1 561 789)	3386.3 (3016.3, 3872.2)	18.7 (12.3, 25.2)
Chad	186 188 (163 947, 212 336)	2637 (2341.5, 2977.6)	671 616 (583 951, 766 547)	2986.1 (2626.4, 3374.1)	13.2 (7.6, 19.2)
Gambia	32 069 (28 087, 36 470)	2675.3 (2364.9, 3019.6)	98 170 (86 416, 112 166)	3147.5 (2790.9, 3570.9)	17.7 (11.6, 24.3)
Ghana	492 912 (431 679, 566 702)	2717 (2388.9, 3095.3)	1 298 237 (1 146 388, 1 480 975)	3191.9 (2831.9, 3629.2)	17.5 (11.9, 23)
Guinea	176 905 (155 492, 201 262)	2699.9 (2384.7, 3056.9)	522 427 (458 689, 595 637)	3116.4 (2762.4, 3527.6)	15.4 (10.5, 21.6)
Guinea‐Bissau	34 492 (30 438, 39 406)	2771 (2466.2, 3133)	82 497 (72 898, 93 903)	3175 (2830, 3586.1)	14.6 (10, 19.5)
Liberia	76 871 (68 024, 87 741)	2719.6 (2417.5, 3084.9)	217 634 (190 832, 248 630)	3120.3 (2755.6, 3539.7)	14.7 (9.3, 21.5)
Mali	261 329 (228 902, 296 476)	2642.6 (2337, 2982.8)	925 333 (810 229, 1 046 858)	3020.5 (2668.7, 3388.2)	14.3 (9.1, 20.1)
Mauritania	68 677 (60 538, 77 883)	2804.8 (2497.2, 3148.8)	179 727 (158 718, 206 618)	3242.1 (2892.2, 3697.1)	15.6 (10.3, 22)
Niger	245 822 (215 666, 281 298)	2589.2 (2299.9, 2935.6)	930 552 (808 207, 1 068 045)	2868.9 (2518.2, 3247.4)	10.8 (4.6, 18.1)
Nigeria	3 221 344 (2 890 157, 3 623 286)	2979.8 (2692.8, 3325.7)	10 274 381 (9 223 246, 11 567 765)	3441.6 (3109.9, 3843)	15.5 (14.3, 16.8)
Sao Tome and Principe	4354 (3826, 4980)	2817.4 (2504.9, 3198.6)	9057 (8056, 10 367)	3303.8 (2961.2, 3754.3)	17.3 (10.8, 22.3)
Senegal	256 237 (225 455, 293 042)	2752.8 (2443.7, 3109.5)	639 709 (562 967, 722 346)	3181.7 (2814.6, 3573.8)	15.6 (9.2, 22.4)
Sierra Leone	129 308 (114 065, 146 017)	2761.8 (2447.5, 3108.5)	351 083 (310 810, 400 350)	3154.1 (2812.2, 3578.2)	14.2 (8.7, 20.6)
Togo	128 752 (112 995, 146 573)	2816.6 (2497.1, 3176.7)	328 123 (291 095, 371 781)	3223.7 (2875.3, 3631)	14.5 (9.6, 19.9)
Taiwan	722 118 (641 844, 815 050)	3225.5 (2861.1, 3647.8)	594 276 (539 650, 656 444)	3786.8 (3350.4, 4279.3)	17.4 (12.2, 22.3)
Turkey	1 854 897 (1 629 145, 2 172 211)	2616.1 (2310.3, 3042.5)	2 470 750 (2 186 694, 2 781 533)	3196.7 (2815.8, 3615.9)	22.2 (16.4, 28.5)
Côte d'Ivoire	405 955 (358 702, 462 503)	2781.3 (2471.5, 3149.9)	1 069 476 (943 844, 1 223 212)	3198.6 (2837.7, 3641.6)	15 (10.1, 21.1)

**TABLE 3:** Incident cases of acne vulgaris in 1990 and 2021 and the percentage change in the age‐standardised rates (ASRs) per 100,000, by location (generated from data available from http://ghdx.healthdata.org/gbd‐results‐tool).

Location_name	1990_No (95% UI)	1990_ASRs_per_100 000 (95% UI)	2021_No (95% UI)	2021_ASRs_per_100 000 (95% UI)	Percentage_change_in_the_ASRs_per_100 000
Bolivia (Plurinational State of)	133 326 (111 451, 157 627)	1619 (1371, 1899)	230 680 (194 707, 273 375)	1843.5 (1553.8, 2187.1)	13.9 (5.5, 21.1)
Ecuador	217 943 (183 315, 258 333)	1676.7 (1421.3, 1975.2)	355 610 (301 115, 419 105)	1892.5 (1601.5, 2230)	12.9 (5.5, 20.6)
Peru	509 604 (437 010, 594 946)	1823 (1572, 2117)	657 960 (558 941, 768 325)	1883.8 (1589.9, 2210.1)	3.3 (−4.4, 11.2)
Australia	266 350 (235 816, 304 001)	1679.8 (1471.8, 1934.2)	371 869 (328 226, 425 427)	1825.4 (1587.6, 2120.2)	8.7 (2.4, 14.7)
New Zealand	58 910 (52 932, 65 565)	1793 (1589.6, 2016.6)	76 458 (68 858, 85 644)	1807.1 (1609.3, 2050.9)	0.8 (−4.5, 6.1)
Antigua and Barbuda	854 (723, 1023)	1287 (1090.3, 1541.6)	1085 (926, 1291)	1459 (1230.4, 1754)	13.4 (7.2, 20.5)
Bahamas	4042 (3427, 4833)	1325.4 (1120, 1588.4)	5702 (4837, 6850)	1480 (1248.5, 1781.2)	11.7 (5.3, 19.4)
Barbados	3281 (2777, 3882)	1289.8 (1085.9, 1533.1)	3108 (2629, 3698)	1408.6 (1174.8, 1696.8)	9.2 (2.4, 17.9)
Belize	3040 (2543, 3657)	1208.2 (1025.3, 1439.9)	6799 (5715, 8146)	1369.7 (1154.3, 1643.1)	13.4 (6.5, 19.7)
Bermuda	632 (543, 733)	1330 (1119.6, 1578.7)	568 (493, 668)	1481.5 (1270.7, 1768.4)	11.4 (4.8, 19.4)
Cuba	131 329 (113 396, 153 689)	1232.2 (1043.2, 1469.8)	104 104 (89 608, 122 010)	1364.9 (1159, 1618.5)	10.8 (4.7, 18.1)
Dominica	1098 (914, 1312)	1251.5 (1046.5, 1488.5)	911 (768, 1079)	1432.5 (1204, 1705.2)	14.5 (6.6, 22.6)
Dominican Republic	109 261 (91 768, 131 954)	1208 (1019.8, 1451.4)	149 297 (125 778, 176 588)	1378.3 (1156.7, 1635.4)	14.1 (6.7, 22.6)
Grenada	1294 (1084, 1544)	1221.6 (1033.4, 1448.2)	1306 (1109, 1529)	1406.8 (1181.8, 1663.5)	15.2 (8.8, 22)
Guyana	12 101 (10 156, 14 633)	1224.1 (1034, 1471.1)	10 857 (9079, 12 939)	1398.6 (1164.9, 1673.3)	14.3 (6.7, 20.8)
Haiti	94 829 (78 913, 114 616)	1194.8 (1006.1, 1430.7)	198 250 (165 162, 235 133)	1345.8 (1123.8, 1594.1)	12.6 (5.5, 21.3)
Jamaica	36 511 (30 454, 43 256)	1242.4 (1043, 1466.3)	36 307 (31 158, 42 843)	1391.5 (1182.1, 1656.7)	12 (4.2, 20)
Puerto Rico	50 139 (42 621, 58 941)	1297.5 (1105.3, 1522.3)	33 892 (29 258, 40 056)	1455 (1231.4, 1741.2)	12.1 (5.5, 18.8)
Saint Kitts and Nevis	630 (531, 755)	1270.5 (1077.5, 1514.5)	655 (563, 760)	1439.3 (1211.2, 1703)	13.3 (6, 20.2)
Saint Lucia	2180 (1818, 2586)	1238.4 (1041.9, 1459.7)	1923 (1642, 2267)	1401.4 (1168.6, 1685.3)	13.2 (6, 20.6)
Saint Vincent and the Grenadines	1788 (1498, 2141)	1218.4 (1030.5, 1445.1)	1425 (1216, 1719)	1379.8 (1170.1, 1670.8)	13.2 (5.5, 20.3)
Suriname	5669 (4749, 6766)	1237.9 (1038.9, 1479.5)	7724 (6533, 9242)	1382 (1166.5, 1654.2)	11.6 (3.6, 18.8)
Trinidad and Tobago	17 612 (15 013, 21 091)	1276.3 (1092.4, 1523.6)	16 143 (13 752, 19 202)	1429.6 (1206.8, 1713.7)	12 (6.5, 18.6)
United States Virgin Islands	1503 (1283, 1799)	1306.1 (1119.1, 1558)	852 (730, 1007)	1460.1 (1239.1, 1736.2)	11.8 (5.1, 19.5)
Armenia	30 034 (25 764, 34 745)	862.8 (737.5, 1002.6)	21 762 (18 673, 25 231)	950.9 (811.4, 1108.2)	10.2 (4, 16.8)
Azerbaijan	72 867 (62 876, 85 772)	889.1 (766.9, 1048.2)	87 937 (76 506, 101 832)	962.7 (833.9, 1118.6)	8.3 (1.4, 14.9)
Georgia	46 267 (39 895, 53 777)	899.4 (770.8, 1049)	24 862 (21 616, 28 660)	952.7 (824.7, 1099.4)	5.9 (1, 13.3)
Kazakhstan	155 971 (133 170, 182 025)	888.1 (759.9, 1035.8)	168 232 (144 140, 196 807)	969.8 (833.2, 1132.5)	9.2 (3.8, 16.3)
Kyrgyzstan	45 318 (38 585, 52 774)	878.4 (751.7, 1018.4)	67 954 (58 144, 79 722)	945.2 (812.5, 1104.4)	7.6 (1.9, 14.3)
Mongolia	23 867 (20 118, 28 639)	857.7 (729.8, 1019.8)	30 163 (25 521, 35 673)	953.4 (811, 1115.7)	11.2 (5, 18.1)
Tajikistan	56 574 (48 073, 66 604)	865 (740.2, 1011.7)	103 250 (87 456, 120 860)	934 (794.4, 1089.4)	8 (1.4, 14.8)
Turkmenistan	39 956 (34 209, 46 653)	883.4 (760.4, 1026.3)	51 143 (44 204, 59 703)	967.2 (834.8, 1131.2)	9.5 (2.9, 17)
Uzbekistan	219 469 (185 301, 258 949)	870.1 (739, 1020.7)	312 199 (268 211, 362 312)	965.2 (826.9, 1121.4)	10.9 (4.9, 17.7)
Albania	28 123 (25 385, 31 491)	735.8 (664.3, 823.8)	16 708 (15 178, 18 414)	826.6 (745.2, 918)	12.3 (6.3, 18.1)
Bosnia and Herzegovina	32 574 (29 637, 35 877)	726.7 (658.7, 804)	18 325 (16 724, 20 188)	838.6 (759.8, 934.3)	15.4 (9.1, 22)
Bulgaria	56 932 (51 886, 62 572)	764.3 (694.7, 843.2)	36 124 (32 903, 39 549)	867.4 (784.6, 956.8)	13.5 (7.7, 20.6)
Croatia	30 829 (27 880, 34 190)	743.3 (667.9, 826.7)	22 457 (20 355, 24 885)	835.3 (750.7, 933.1)	12.4 (6.7, 18.1)
Czechia	72 541 (65 415, 80 875)	743.7 (669.7, 829.5)	57 279 (51 929, 64 165)	843.9 (760.8, 951.3)	13.5 (7.2, 20.9)
Hungary	69 822 (63 389, 77 958)	740.8 (672.1, 827.5)	50 668 (45 868, 55 603)	838.6 (752.4, 928.5)	13.2 (7.4, 19.1)
Montenegro	4704 (4247, 5266)	750.9 (676.7, 842.7)	3977 (3606, 4426)	846.5 (764, 946.7)	12.7 (7.2, 19)
North Macedonia	14 890 (13 418, 16 674)	736.2 (662.5, 825.5)	12 426 (11 339, 13 795)	830.6 (750.4, 933.1)	12.8 (6.8, 20.4)
Poland	277 115 (254 695, 303 251)	777.2 (713.9, 850.7)	208 276 (196 149, 220 608)	842.9 (789.7, 898.3)	8.4 (3.5, 14.3)
Romania	163 820 (147 910, 182 823)	712.2 (643.9, 795.3)	105 735 (96 013, 117 480)	821.7 (742.5, 918.5)	15.4 (9.5, 22.2)
Serbia	65 657 (59 004, 73 314)	745.4 (668.1, 834.1)	55 372 (50 444, 61 147)	838.4 (757.7, 931.9)	12.5 (6.5, 19.2)
Slovakia	38 805 (35 023, 43 344)	744.5 (672, 830.8)	29 737 (27 212, 32 780)	841.5 (763.8, 935.3)	13 (6.9, 19.4)
Slovenia	13 546 (12 268, 14 992)	766.7 (690.9, 852.2)	10 797 (9768, 11 938)	857.2 (769.7, 955.1)	11.8 (6.4, 18.8)
Colombia	464 315 (389 246, 553 468)	1178.3 (992.1, 1399.8)	590 366 (502 237, 685 504)	1335.7 (1122, 1558.3)	13.4 (4.5, 22.1)
Costa Rica	43 748 (37 513, 51 965)	1206.1 (1039.1, 1425.1)	57 481 (48 739, 67 743)	1362.7 (1148, 1617)	13 (6.5, 19.8)
El Salvador	83 481 (69 326, 99 736)	1180.5 (993.6, 1397.6)	89 288 (75 404, 105 270)	1344.1 (1132.3, 1587.8)	13.9 (6.4, 21.1)
Guatemala	130 584 (108 034, 156 347)	1146.4 (965.8, 1349.6)	243 443 (205 529, 289 158)	1317.1 (1111.4, 1572.4)	14.9 (8.7, 22.1)
Honduras	75 142 (62 077, 90 586)	1167.2 (981.8, 1387.9)	158 656 (132 683, 187 625)	1318.5 (1102.3, 1555.6)	13 (6.3, 20.5)
Mexico	1 375 347 (1 202 643, 1 599 522)	1215.3 (1070.1, 1403)	1 743 063 (1 534 187, 2 008 993)	1356.9 (1192.5, 1567.8)	11.7 (10, 13.4)
Nicaragua	62 404 (51 848, 74 630)	1157.3 (977.7, 1369.6)	96 269 (81 680, 113 844)	1320.8 (1119.5, 1561.7)	14.1 (6.5, 22.8)
Panama	36 403 (31 154, 42 845)	1251.5 (1074.4, 1466.7)	60 443 (51 105, 71 665)	1415.6 (1195, 1680.9)	13.1 (6.6, 19.7)
Venezuela (Bolivarian Republic of)	290 960 (245 803, 345 811)	1233.1 (1049.4, 1455.4)	341 367 (290 109, 411 434)	1368.2 (1160.8, 1650.8)	11 (3.3, 18.3)
Angola	204 170 (170 157, 247 308)	1578.7 (1327.6, 1887.1)	814 582 (676 963, 992 174)	1865.2 (1576.1, 2234.3)	18.2 (11.5, 24.4)
Central African Republic	52 548 (43 591, 63 128)	1568.4 (1312, 1868.5)	127 151 (105 926, 153 952)	1772.1 (1499, 2124.1)	13 (7, 21)
Congo	52 971 (44 198, 63 514)	1650.6 (1400.8, 1957.3)	127 876 (108 141, 154 558)	1910.2 (1625.2, 2286)	15.7 (8.6, 23.9)
Democratic Republic of the Congo	763 835 (640 466, 919 968)	1589.4 (1349, 1889.7)	2 143 044 (1 788 534, 2 574 204)	1799.6 (1522.1, 2139)	13.2 (5.9, 22.9)
Equatorial Guinea	8268 (6872, 9789)	1573.9 (1325.9, 1841.5)	40 424 (33 796, 48 497)	1924.8 (1624.2, 2290.3)	22.3 (12.9, 30.6)
Gabon	19 895 (16 581, 23 674)	1637.8 (1383.3, 1929.9)	43 052 (36 043, 51 772)	1945.8 (1641.8, 2323.5)	18.8 (11, 27.1)
China	20 793 409 (18 565 210, 23 388 070)	1632.4 (1445.7, 1843.8)	19 912 141 (17 768 188, 22 495 198)	1915.8 (1694.7, 2182)	17.4 (15.5, 19.1)
Democratic People's Republic of Korea	332 595 (292 379, 386 069)	1607.9 (1403.1, 1876.3)	387 055 (339 726, 443 696)	1817.6 (1568.1, 2103.8)	13 (7.4, 18.6)
Belarus	79 907 (69 780, 92 413)	886 (768.7, 1032.1)	60 380 (51 948, 69 854)	979.7 (838.8, 1139.5)	10.6 (3.7, 17.3)
Estonia	11 831 (10 197, 13 578)	891.7 (764.8, 1031.6)	8589 (7354, 9995)	989.5 (841.6, 1156.3)	11 (4.4, 18.1)
Latvia	19 343 (16 849, 22 269)	894.2 (772.9, 1041.8)	11 704 (10 212, 13 438)	991.2 (858, 1148.7)	10.8 (4.8, 16.2)
Lithuania	29 055 (25 094, 33 843)	896.1 (765.2, 1057.1)	16 860 (14 796, 19 293)	998.9 (864.6, 1161.4)	11.5 (5.9, 18.4)
Republic of Moldova	37 514 (32 253, 43 663)	880.3 (756.6, 1024.6)	22 733 (19 678, 26 227)	971.2 (830.7, 1140.6)	10.3 (4.8, 16.9)
Russian Federation	1 174 877 (1 046 487, 1 337 072)	904.6 (801.8, 1036.5)	1 009 510 (900 987, 1 144 565)	988 (876.1, 1123.1)	9.2 (8.1, 10.3)
Ukraine	395 412 (347 607, 448 814)	893.3 (779.9, 1020.6)	278 042 (244 816, 313 846)	968.7 (847.3, 1102.3)	8.4 (3.8, 14.1)
Burundi	132 701 (111 566, 157 520)	1918.9 (1635.6, 2249.9)	383 854 (320 103, 456 270)	2166.2 (1835.7, 2552.7)	12.9 (4.9, 21.9)
Comoros	12 059 (10 230, 14 403)	1941.1 (1667.3, 2290.3)	19 399 (16 591, 22 935)	2258.6 (1937.6, 2665.5)	16.4 (8.9, 24)
Djibouti	10 679 (8999, 12 712)	1886.8 (1606.2, 2224.1)	30 869 (26 278, 36 411)	2185.1 (1861.6, 2574.8)	15.8 (8.6, 23.3)
Eritrea	86 769 (73 534, 103 705)	1900.4 (1633.3, 2244.4)	181 005 (154 610, 215 242)	2212.7 (1900.1, 2616.1)	16.4 (9.8, 23.8)
Ethiopia	1 242 568 (1 069 851, 1 445 452)	1864.5 (1630.3, 2137.9)	3 197 020 (2 771 545, 3 696 802)	2181.5 (1907.3, 2507.3)	17 (12.9, 21)
Kenya	612 003 (530 112, 705 016)	1907.9 (1681.3, 2172.9)	1 495 708 (1 303 748, 1 715 039)	2211.1 (1940.7, 2519.8)	15.9 (14.1, 18)
Madagascar	299 854 (251 028, 356 159)	1928.1 (1635.9, 2262.7)	839 004 (708 947, 990 564)	2199.3 (1877.5, 2575.9)	14.1 (6.9, 22.7)
Malawi	238 313 (201 091, 282 569)	1902.1 (1622.5, 2237.3)	615 838 (516 797, 724 214)	2199 (1868.4, 2564.8)	15.6 (9.6, 23.1)
Mozambique	325 046 (271 911, 384 005)	1868.1 (1585.7, 2180.2)	928 398 (783 901, 1 110 659)	2169.1 (1856.7, 2558.8)	16.1 (9.3, 23.7)
Rwanda	181 444 (153 248, 216 333)	1953.9 (1676.4, 2303.3)	377 733 (320 512, 447 317)	2244.2 (1914.9, 2645.5)	14.9 (7.5, 22.3)
Somalia	195 201 (162 200, 233 986)	1791.1 (1523.4, 2112.8)	578 128 (484 543, 689 412)	1985.6 (1682.1, 2347.4)	10.9 (3.7, 20.8)
South Sudan	148 284 (125 296, 176 254)	1890.8 (1614.1, 2223.3)	293 190 (244 427, 347 137)	2129.9 (1802.7, 2493.1)	12.6 (5.1, 21.8)
Uganda	422 536 (355 261, 500 516)	1867 (1586.9, 2187.8)	1 333 289 (1 130 341, 1 568 868)	2198.6 (1883.8, 2552.9)	17.8 (10.8, 25.2)
United Republic of Tanzania	682 297 (576 049, 805 708)	1978.2 (1690.5, 2303.6)	1 710 289 (1 449 829, 2 019 589)	2224.6 (1908.7, 2601.5)	12.5 (5.9, 22.4)
Zambia	213 006 (180 260, 251 240)	1940.4 (1666.8, 2265.9)	594 585 (500 185, 716 011)	2260.9 (1920.9, 2693.7)	16.5 (9.2, 24.3)
Brunei Darussalam	5814 (5027, 6717)	1975.3 (1703.3, 2285.9)	9242 (8183, 10 506)	2171.3 (1885.5, 2514.2)	9.9 (2.8, 15.6)
Japan	2 310 513 (2 095 628, 2 572 726)	2063.1 (1842.5, 2318.8)	1 739 507 (1 584 396, 1 919 393)	2241.1 (1992.5, 2518.8)	8.6 (7.2, 10.3)
Republic of Korea	990 296 (860 837, 1 136 264)	1996.6 (1722.7, 2297.2)	745 807 (674 215, 837 608)	2175.4 (1908.3, 2512.3)	9 (3.1, 14.9)
Singapore	61 897 (54 709, 69 975)	1997.4 (1717.4, 2312.7)	81 665 (74 003, 92 389)	2201.7 (1915.1, 2583.2)	10.2 (3.8, 16.1)
Canada	343 693 (317 500, 372 869)	1366.3 (1251, 1500.9)	427 425 (395 590, 465 796)	1484.9 (1362.1, 1643.1)	8.7 (3.8, 14)
Greenland	717 (663, 775)	1318.9 (1210.2, 1448.2)	708 (652, 770)	1464 (1337.5, 1607.4)	11 (6.1, 16)
United States of America	3 341 296 (3 128 443, 3 620 866)	1429.3 (1327.7, 1561.9)	4 440 169 (4 211 083, 4 698 890)	1618.1 (1525.2, 1725.8)	13.2 (8, 19.5)
Afghanistan	170 201 (143 903, 200 924)	1286.8 (1101.3, 1500.9)	650 679 (540 193, 764 558)	1555.1 (1306.8, 1817.1)	20.9 (13, 29.3)
Algeria	455 595 (386 148, 532 156)	1365.4 (1176.3, 1581.9)	710 686 (607 287, 830 353)	1658.9 (1414.8, 1924)	21.5 (14.1, 30)
Bahrain	7205 (6277, 8302)	1393.3 (1209.6, 1616.4)	21 814 (19 056, 25 034)	1679.7 (1449.9, 1967.1)	20.5 (13.4, 29.2)
Egypt	980 733 (842 613, 1 162 657)	1437.7 (1245.7, 1691.2)	2 023 669 (1 735 428, 2 388 553)	1699 (1460.7, 1994)	18.2 (11.6, 25.7)
Iran (Islamic Republic of)	1 002 554 (881 767, 1 137 953)	1347.8 (1194.4, 1515.4)	1 259 927 (1 116 877, 1 426 114)	1664.5 (1468.8, 1898.7)	23.5 (21.6, 25.7)
Iraq	303 563 (258 000, 357 832)	1251.7 (1073.9, 1459.9)	768 209 (647 751, 898 208)	1564.6 (1324.4, 1824)	25 (17.5, 34.8)
Jordan	70 888 (60 174, 82 861)	1375.5 (1180.3, 1597.9)	241 565 (206 448, 286 268)	1655.1 (1415.3, 1963.7)	20.3 (13.4, 28.4)
Kuwait	25 651 (22 028, 29 809)	1420.9 (1224.7, 1654)	61 794 (53 708, 71 085)	1720 (1460.7, 2020.1)	21.1 (13.8, 30.2)
Lebanon	46 028 (39 577, 53 345)	1354.1 (1169.8, 1565.4)	82 520 (71 029, 95 532)	1657.6 (1415.6, 1929)	22.4 (15.6, 30.1)
Libya	78 704 (66 632, 92 430)	1348.9 (1156.2, 1566)	113 086 (98 073, 130 007)	1670.4 (1438.7, 1938.5)	23.8 (16.4, 31.9)
Morocco	418 911 (357 537, 491 475)	1324.2 (1138.6, 1543)	587 855 (503 521, 685 728)	1594 (1359.7, 1864.3)	20.4 (12.7, 28.6)
Oman	31 521 (27 052, 37 190)	1367.5 (1182.9, 1600.3)	70 082 (60 615, 81 339)	1702.2 (1454.7, 2001.3)	24.5 (18.1, 32)
Palestine	35 422 (30 236, 41 647)	1322.2 (1141.1, 1542.3)	105 502 (89 936, 124 429)	1626.6 (1395.7, 1905.9)	23 (15.6, 31.7)
Qatar	5724 (5000, 6562)	1405.2 (1214.3, 1624.8)	33 713 (29 909, 38 198)	1704.2 (1478.3, 1987.6)	21.3 (14.6, 28.3)
Saudi Arabia	277 698 (237 183, 327 314)	1395.9 (1200.9, 1634.8)	563 611 (495 209, 648 898)	1732 (1496.7, 2021.2)	24.1 (16.6, 32.6)
Sudan	332 553 (282 630, 393 752)	1288.1 (1110.1, 1511.1)	907 781 (770 659, 1 070 801)	1609.6 (1376.8, 1887.6)	25 (15.8, 33.4)
Syrian Arab Republic	230 167 (196 181, 268 284)	1319.2 (1137, 1522.9)	280 879 (239 589, 327 556)	1627.6 (1397.6, 1896.5)	23.4 (14.8, 31.1)
Tunisia	141 122 (121 043, 163 747)	1356.4 (1172.5, 1563.7)	171 949 (147 584, 202 537)	1655.4 (1414.9, 1957)	22 (15.1, 29.7)
United Arab Emirates	24 443 (21 570, 28 276)	1412.6 (1233.9, 1648.5)	98 203 (86 805, 113 231)	1733.5 (1485.7, 2042.1)	22.7 (16.2, 29.2)
Yemen	231 943 (195 213, 274 937)	1270.1 (1086.9, 1481.5)	711 829 (592 632, 839 011)	1576.8 (1335.9, 1836.4)	24.1 (16.3, 32.5)
American Samoa	825 (713, 961)	1433.9 (1243.8, 1666.8)	896 (766, 1074)	1588.9 (1369.8, 1874.2)	10.8 (5.4, 16.4)
Cook Islands	325 (280, 383)	1427.7 (1238.7, 1667)	256 (223, 298)	1608.2 (1398.5, 1875.5)	12.6 (7.6, 17.9)
Fiji	12 991 (10 994, 15 322)	1387.2 (1183.5, 1623.2)	15 031 (12 907, 17 658)	1563.9 (1347.3, 1832.3)	12.7 (6.4, 18.3)
Guam	2107 (1830, 2460)	1454.9 (1253, 1717.4)	2278 (1969, 2657)	1623.1 (1395.4, 1898.3)	11.6 (6.5, 16.7)
Kiribati	1140 (985, 1337)	1322.9 (1145.1, 1548.3)	2064 (1743, 2439)	1461.9 (1243.5, 1718.3)	10.5 (5.3, 17.3)
Marshall Islands	856 (721, 1013)	1352.2 (1160.3, 1570)	977 (836, 1152)	1516.9 (1301.1, 1781.4)	12.2 (7.2, 17.9)
Micronesia (Federated States of)	1931 (1634, 2293)	1371.6 (1185.3, 1606.3)	1813 (1556, 2124)	1526.1 (1311.1, 1780.1)	11.3 (6.2, 16)
Nauru	170 (145, 198)	1392.5 (1200.5, 1615.5)	206 (178, 241)	1545.6 (1340.6, 1796)	11 (4.9, 17.2)
Niue	37 (32, 43)	1405.4 (1221.6, 1639.1)	25 (22, 29)	1578.7 (1370, 1839.7)	12.3 (6.8, 18.4)
Northern Mariana Islands	696 (611, 799)	1492.6 (1294.3, 1742.1)	739 (637, 865)	1606.7 (1392.9, 1878.7)	7.6 (2.8, 14)
Palau	255 (221, 298)	1442.4 (1250.8, 1683.4)	230 (199, 268)	1597.9 (1367.9, 1877.6)	10.8 (5.2, 16.3)
Papua New Guinea	66 369 (56 789, 78 593)	1295.5 (1119.2, 1519.9)	172 572 (147 812, 202 156)	1436.8 (1238.4, 1676.7)	10.9 (6, 17)
Samoa	3167 (2687, 3757)	1388.7 (1194.1, 1624.8)	3843 (3270, 4564)	1537.3 (1319.1, 1809.8)	10.7 (4.3, 15.9)
Solomon Islands	5886 (5009, 6985)	1297.5 (1118.6, 1516.8)	12 002 (10 202, 14 163)	1449.7 (1242.5, 1698.9)	11.7 (6.6, 17.3)
Tokelau	25 (22, 29)	1359.9 (1168, 1579.5)	23 (20, 27)	1542.4 (1331.5, 1792.3)	13.4 (7.3, 19.9)
Tonga	1804 (1541, 2138)	1387.5 (1202.9, 1621.8)	1902 (1613, 2232)	1552 (1330.2, 1810.9)	11.9 (6.3, 18.4)
Tuvalu	132 (114, 156)	1353.8 (1167.6, 1593.2)	204 (174, 243)	1521.1 (1300.9, 1808.1)	12.4 (6.7, 18.5)
Vanuatu	2519 (2134, 2979)	1324.4 (1137.1, 1551.8)	5475 (4637, 6433)	1483.4 (1266.3, 1733.5)	12 (6.1, 18.6)
Bangladesh	1 383 069 (1 176 901, 1 614 254)	1006 (866.1, 1157.9)	2 127 281 (1 823 115, 2 469 388)	1194 (1022, 1387.2)	18.7 (12.1, 24.8)
Bhutan	8336 (7057, 9744)	971.7 (832.8, 1128.8)	8977 (7760, 10 341)	1154.9 (989.8, 1337.3)	18.9 (12, 25.7)
India	10 531 241 (9 243 847, 11 964 680)	1038.5 (918.3, 1172.6)	18 933 221 (16 726 830, 21 419 551)	1240.8 (1091.9, 1409.2)	19.5 (18, 21)
Nepal	227 876 (191 353, 269 402)	953.2 (806.7, 1111.9)	392 745 (330 818, 458 323)	1113.1 (940.2, 1303)	16.8 (10.8, 23.9)
Pakistan	1 344 198 (1 171 271, 1 541 264)	946.2 (834.9, 1073.9)	3 170 249 (2 792 852, 3 639 891)	1115.1 (987.4, 1273.5)	17.9 (13.8, 22)
Cambodia	183 715 (157 669, 214 793)	1427.5 (1243.9, 1645.7)	295 062 (256 107, 342 304)	1613.5 (1400.7, 1870.8)	13 (7.7, 18.8)
Indonesia	3 507 001 (3 082 243, 4 002 498)	1518.5 (1344.1, 1720.1)	4 689 631 (4 180 795, 5 315 585)	1741.3 (1544.5, 1982.9)	14.7 (13, 16.5)
Lao People's Democratic Republic	73 456 (63 042, 85 860)	1407.8 (1226.5, 1629.1)	129 579 (111 930, 150 253)	1609.6 (1392.4, 1869.7)	14.3 (9.2, 21.1)
Malaysia	318 856 (275 565, 371 051)	1513.2 (1316.4, 1751.2)	533 821 (466 099, 617 605)	1717.2 (1487.8, 2001)	13.5 (8.3, 18.9)
Maldives	3974 (3394, 4729)	1400.2 (1206.5, 1643.3)	7092 (6249, 8118)	1633.9 (1409.5, 1902.2)	16.7 (10.2, 22.7)
Mauritius	18 720 (16 198, 22 043)	1499.5 (1300.4, 1756.9)	17 173 (15 094, 19 848)	1722.8 (1495.7, 2029.1)	14.9 (8.4, 20.5)
Myanmar	715 973 (609 462, 836 734)	1430.1 (1226, 1660.2)	967 530 (837 136, 1 125 506)	1648.5 (1424.8, 1920.6)	15.3 (9.9, 21.4)
Philippines	1 190 781 (1 053 455, 1 365 669)	1501.3 (1337.7, 1707.3)	2 103 674 (1 865 502, 2 400 205)	1679.3 (1490.1, 1915.2)	11.9 (10.7, 13.5)
Seychelles	1318 (1144, 1549)	1551.7 (1350.5, 1812.9)	1561 (1367, 1822)	1728.5 (1503.6, 2034.9)	11.4 (7.3, 17.1)
Sri Lanka	298 889 (258 766, 352 068)	1488.8 (1295.3, 1746.7)	346 809 (301 818, 406 869)	1670.2 (1445.9, 1966.1)	12.2 (7.8, 17.5)
Thailand	963 406 (834 383, 1 118 368)	1445.7 (1254.5, 1674.1)	751 827 (659 588, 867 236)	1594.7 (1367.6, 1868.8)	10.3 (5.3, 17.2)
Timor‐Leste	12 831 (11 097, 15 079)	1418.8 (1232.9, 1661)	29 400 (25 333, 34 798)	1663.6 (1446.5, 1952.5)	17.3 (10.8, 23.4)
Viet Nam	1 239 516 (1 071 320, 1 458 508)	1473.1 (1284.8, 1720.9)	1 521 758 (1 325 186, 1 771 064)	1680.7 (1456.2, 1963.3)	14.1 (8.9, 19.4)
Uruguay	50 577 (44 368, 58 308)	1653.2 (1449.3, 1905.3)	52 607 (46 588, 60 066)	1799.6 (1570.8, 2083.1)	8.9 (3.3, 15)
Argentina	569 012 (498 103, 658 966)	1647.1 (1450.5, 1891.6)	774 436 (680 577, 885 390)	1797.6 (1565.2, 2074.6)	9.1 (3.7, 14.3)
Chile	236 829 (207 059, 271 015)	1655.3 (1438.8, 1897)	296 088 (263 837, 337 670)	1804.7 (1583.5, 2087.4)	9 (3.2, 14.8)
Botswana	29 990 (24 815, 35 964)	1655.9 (1391.3, 1957.6)	49 938 (42 326, 58 973)	1929.8 (1632.8, 2282.8)	16.5 (10.6, 23.8)
Eswatini	17 993 (14 870, 21 750)	1616.1 (1360.5, 1924.6)	27 028 (22 595, 32 607)	1882.6 (1584, 2257.5)	16.5 (9.2, 23.2)
Lesotho	32 380 (26 551, 39 239)	1612.6 (1351.8, 1922.8)	43 788 (36 733, 52 164)	1874.6 (1580.5, 2228.6)	16.2 (8.5, 23.5)
Namibia	30 745 (25 794, 37 039)	1658.2 (1406.5, 1980)	56 177 (47 119, 67 820)	1925.3 (1623.8, 2312.1)	16.1 (10.3, 23.9)
South Africa	776 326 (673 353, 903 585)	1702.1 (1482.2, 1974.1)	1 112 402 (978 447, 1 280 901)	1933.7 (1694.4, 2233.9)	13.6 (10.8, 16.9)
Zimbabwe	236 981 (198 006, 286 571)	1645.6 (1397.2, 1959.1)	375 194 (311 343, 457 648)	1848.9 (1551.4, 2229.5)	12.4 (6, 21.2)
Brazil	2 089 259 (1 823 161, 2 410 129)	1132.9 (993.9, 1301)	2 352 439 (2 079 300, 2 681 724)	1262.1 (1105, 1451.3)	11.4 (9.5, 13.5)
Paraguay	59 427 (50 094, 71 650)	1177.7 (1002, 1407.2)	102 674 (86 590, 121 050)	1340.1 (1129.1, 1580.9)	13.8 (5.9, 21.9)
Andorra	1188 (1063, 1339)	2371.3 (2075.8, 2742.5)	1514 (1352, 1718)	2579.8 (2259.8, 3011.9)	8.8 (3.1, 15.2)
Austria	148 443 (132 213, 167 721)	2346.1 (2056, 2711.8)	155 645 (139 154, 174 528)	2541.8 (2214.7, 2928.7)	8.3 (2.3, 13.8)
Belgium	190 850 (168 528, 216 330)	2331 (2012.9, 2705)	215 334 (190 366, 245 385)	2551 (2223.5, 2947.2)	9.4 (3.3, 15.8)
Cyprus	17 565 (15 324, 20 348)	2314 (2007, 2692.6)	25 687 (23 003, 28 757)	2572.4 (2246.7, 2954.9)	11.2 (5.2, 17.4)
Denmark	103 334 (91 837, 117 286)	2381.1 (2074.5, 2767)	112 477 (99 444, 128 115)	2581.3 (2249.4, 2992.9)	8.4 (2.9, 15)
Finland	97 114 (86 431, 110 589)	2357.3 (2066.6, 2724.9)	102 039 (90 316, 116 295)	2567 (2239.8, 2972.8)	8.9 (3, 15.8)
France	1 135 096 (1 004 752, 1 296 523)	2259.4 (1964.4, 2623.5)	1 253 512 (1 098 836, 1 447 791)	2472.8 (2140.2, 2887.6)	9.4 (3.5, 15.7)
Germany	1 553 253 (1 413 532, 1 724 254)	2550.6 (2258.5, 2905.3)	1 510 176 (1 362 078, 1 679 681)	2723 (2405, 3093.7)	6.8 (1.6, 14.2)
Greece	209 887 (183 976, 242 580)	2257.6 (1958.5, 2643.1)	169 439 (149 039, 193 212)	2493.6 (2159.2, 2887.8)	10.5 (4.4, 17.6)
Iceland	5809 (5104, 6655)	2333.6 (2034.9, 2703.3)	7354 (6434, 8443)	2566 (2221.4, 2980.7)	10 (4, 17.6)
Ireland	89 871 (79 044, 104 329)	2332.7 (2057.8, 2697.3)	107 134 (93 623, 123 221)	2583.9 (2244, 2985.5)	10.8 (4.3, 16.9)
Israel	125 126 (108 649, 145 413)	2335.8 (2037.6, 2704)	230 484 (201 849, 267 824)	2547.2 (2230, 2958.1)	9 (3.9, 14.6)
Italy	1 161 730 (1 056 283, 1 286 846)	2392.8 (2138.9, 2696.5)	988 270 (901 472, 1 089 188)	2578.1 (2309.8, 2897.7)	7.7 (5.5, 10.1)
Luxembourg	7235 (6480, 8149)	2409.6 (2105.1, 2779.4)	12 387 (11 042, 14 030)	2621 (2284.3, 3042.8)	8.8 (3.2, 15.4)
Malta	7909 (6910, 9117)	2283.9 (1984.7, 2645.7)	7182 (6443, 8172)	2501 (2184.4, 2938.1)	9.5 (3.7, 15.7)
Monaco	447 (405, 495)	2382.8 (2090.6, 2733.4)	591 (530, 672)	2573.8 (2258.4, 3004.1)	8 (2.1, 14.4)
Netherlands	302 177 (269 753, 338 179)	2354.2 (2044.4, 2701.4)	321 273 (288 219, 365 502)	2575.2 (2261.4, 3000.2)	9.4 (3.9, 15.7)
Norway	87 230 (79 470, 96 646)	2426.7 (2169.5, 2737.8)	110 820 (100 074, 123 610)	2655.6 (2365.3, 3002.9)	9.4 (6.9, 12.5)
Portugal	240 179 (209 651, 273 295)	2439.5 (2113.8, 2798.6)	188 142 (167 874, 211 261)	2667.1 (2330.2, 3063.7)	9.3 (4.1, 14.9)
San Marino	513 (457, 580)	2389.5 (2099.2, 2754.3)	590 (526, 674)	2595.6 (2268.3, 3017.3)	8.6 (3.5, 15.2)
Spain	872 271 (770 700, 1 002 471)	2309 (2026.4, 2674.6)	787 176 (696 463, 894 174)	2518.4 (2189.3, 2907.7)	9.1 (2.8, 15.6)
Sweden	152 932 (138 153, 169 935)	2290.9 (2032.9, 2592.3)	192 308 (172 768, 216 009)	2480 (2200.7, 2819)	8.3 (3.3, 14.4)
Switzerland	130 301 (116 705, 145 733)	2399.7 (2086.1, 2755.4)	156 136 (139 612, 175 878)	2575.6 (2247.1, 2969.1)	7.3 (1.3, 15)
United Kingdom	1 120 474 (1 016 026, 1 243 164)	2390 (2128.7, 2701.7)	1 351 939 (1 222 699, 1 509 752)	2619.9 (2341.7, 2965.5)	9.6 (7.9, 11.4)
Benin	90 437 (77 125, 105 534)	1495.6 (1294.4, 1730.2)	305 408 (256 333, 361 181)	1736.9 (1480.2, 2035.6)	16.1 (9.1, 23.7)
Burkina Faso	179 666 (150 957, 210 286)	1450.2 (1240.9, 1671.2)	500 960 (418 967, 590 977)	1698.2 (1446.6, 1984.4)	17.1 (10.1, 25.7)
Cabo Verde	6819 (5773, 8065)	1507.2 (1291.8, 1760.4)	10 417 (8934, 12 066)	1788.1 (1523.2, 2076.7)	18.6 (10.6, 26.3)
Cameroon	199 690 (167 943, 237 058)	1527 (1301.7, 1786.3)	755 428 (632 851, 890 478)	1810.3 (1535.1, 2115)	18.5 (10.9, 26.7)
Chad	109 256 (92 678, 128 411)	1453.2 (1252.1, 1687.5)	401 935 (333 076, 474 009)	1670.2 (1412.7, 1948.6)	14.9 (7.7, 21.9)
Gambia	18 531 (15 714, 21 746)	1468.2 (1256.6, 1706.8)	55 435 (46 419, 65 380)	1732.7 (1468.7, 2027.5)	18 (9.7, 26.4)
Ghana	280 992 (237 219, 332 267)	1478.9 (1263.6, 1733.4)	723 138 (613 268, 855 141)	1743.2 (1486.7, 2051.6)	17.9 (10.5, 24.4)
Guinea	103 441 (86 002, 121 388)	1474.1 (1241.6, 1713.3)	305 308 (253 753, 358 755)	1721.5 (1454.8, 2002.3)	16.8 (10.5, 26.5)
Guinea‐Bissau	19 927 (16 875, 23 557)	1501.6 (1292.6, 1748)	47 290 (39 839, 56 450)	1741.6 (1488.4, 2054.5)	16 (9.7, 22.5)
Liberia	44 168 (37 382, 52 266)	1482.8 (1267, 1734.1)	123 936 (103 855, 145 829)	1722.7 (1459.5, 2007.6)	16.2 (8.6, 25.1)
Mali	154 204 (128 752, 182 232)	1453.6 (1238.7, 1698.3)	542 710 (456 177, 628 536)	1680.5 (1435.2, 1928.2)	15.6 (8.9, 22.1)
Mauritania	38 703 (33 075, 45 179)	1509.1 (1303.7, 1747.2)	102 260 (86 038, 121 291)	1765.5 (1504.1, 2067.4)	17 (9.9, 24.8)
Niger	146 571 (122 776, 173 971)	1432.1 (1218.3, 1675.7)	560 909 (471 664, 667 367)	1623.6 (1388.5, 1905.1)	13.4 (5.4, 22.8)
Nigeria	1 758 955 (1 534 856, 2 023 968)	1571.9 (1383.7, 1796.4)	5 715 581 (4 960 937, 6 638 399)	1831.8 (1606.9, 2104.8)	16.5 (15, 18.2)
Sao Tome and Principe	2518 (2115, 2964)	1516.5 (1300.3, 1761.4)	4989 (4279, 5861)	1784.6 (1544.4, 2082)	17.7 (9.5, 24.7)
Senegal	147 671 (125 565, 175 211)	1495.3 (1288.3, 1753.2)	359 895 (304 011, 421 772)	1742.9 (1485, 2023.9)	16.6 (8.2, 25.8)
Sierra Leone	72 339 (61 624, 84 479)	1496.6 (1284.3, 1737)	195 369 (165 325, 229 903)	1734.2 (1476.7, 2032.2)	15.9 (8.4, 25)
Togo	73 592 (63 047, 86 111)	1516.7 (1311.5, 1752.2)	185 146 (156 222, 218 165)	1757.7 (1495.5, 2056.4)	15.9 (9.4, 22.3)
Taiwan	359 142 (312 123, 416 069)	1627.4 (1417.2, 1884)	274 618 (246 621, 310 881)	1900.8 (1653.8, 2220.7)	16.8 (11.4, 22.2)
Turkey	982 177 (839 510, 1 163 802)	1366.5 (1175.9, 1607.8)	1 264 876 (1 081 215, 1 459 078)	1679 (1426, 1947)	22.9 (15.9, 30.2)
Côte d'Ivoire	230 074 (196 106, 271 143)	1501.4 (1291.2, 1760.2)	607 730 (513 130, 710 547)	1748.4 (1486.9, 2028.9)	16.4 (10, 24.2)

**TABLE 4:** DALYs due to acne vulgaris in 1990 and 2021 and the percentage change in the age‐standardised rates (ASRs) per 100,000, by location (generated from data available from http://ghdx.healthdata.org/gbd‐results‐tool).

Location_name	1990_No (95% UI)	1990_ASRs_per_100 000 (95% UI)	2021_No (95% UI)	2021_ASRs_per_100 000 (95% UI)	Percentage_change_in_the_ASRs_per_100 000
Bolivia (Plurinational State of)	5069 (3142, 8194)	64.3 (40.1, 104)	9227 (5570, 14 780)	72.8 (43.9, 116.8)	13.3 (4.1, 22.2)
Ecuador	8593 (5402, 13 266)	67.5 (42.5, 104.5)	14 330 (8685, 22 548)	75.9 (46, 119.4)	12.4 (4.2, 21.5)
Peru	20 013 (12 246, 31 812)	72.9 (44.7, 115.6)	27 095 (16 282, 42 693)	75.4 (45.3, 118.8)	3.4 (−5.1, 12)
Australia	11 125 (6960, 17 675)	67.2 (42, 107)	15 301 (9516, 23 854)	73.3 (45.5, 114.5)	9.1 (2, 17.5)
New Zealand	2464 (1533, 3955)	71.4 (44.7, 114.8)	3150 (1927, 4900)	72.3 (44.1, 112.3)	1.2 (−5.4, 7.1)
Antigua and Barbuda	35 (21, 56)	52.2 (31.8, 83.2)	46 (29, 74)	59.1 (36.5, 93.3)	13.2 (4.9, 22.8)
Bahamas	169 (105, 276)	54.4 (33.7, 88.7)	240 (148, 388)	60.4 (37.1, 97.6)	11 (2.2, 21)
Barbados	138 (85, 220)	52.3 (32.2, 83.6)	132 (82, 211)	56.5 (35.4, 91.1)	7.9 (−1.1, 17.9)
Belize	116 (69, 184)	48 (28.6, 76.4)	279 (171, 443)	54.4 (33.5, 86.4)	13.4 (4.3, 22.6)
Bermuda	28 (17, 44)	54.8 (33.5, 87.3)	24 (15, 39)	60.7 (37.2, 97.8)	10.7 (1.9, 19.4)
Cuba	5897 (3635, 9332)	49.3 (30.7, 78)	4341 (2619, 6873)	54.3 (32.5, 86.1)	10 (0.6, 19.5)
Dominica	45 (27, 72)	50.3 (30.6, 81)	38 (23, 60)	57.6 (35, 90.7)	14.4 (5.5, 24.3)
Dominican Republic	4378 (2656, 6877)	48.1 (29.1, 75.8)	6136 (3735, 9630)	54.8 (33.3, 85.9)	13.8 (4.8, 25)
Grenada	50 (30, 78)	48.5 (29.7, 75.5)	55 (35, 89)	56.4 (35.3, 90.8)	16.2 (7.3, 27.6)
Guyana	483 (301, 776)	48.6 (30.3, 78)	445 (274, 695)	55.6 (34.3, 87.2)	14.4 (5.3, 24)
Haiti	3558 (2187, 5714)	46.8 (28.6, 75.2)	7761 (4778, 12 315)	52.7 (32.5, 83.7)	12.5 (2.4, 23.2)
Jamaica	1458 (895, 2372)	49.7 (30.6, 81)	1532 (930, 2430)	55.6 (33.7, 87.8)	11.8 (2.7, 22.4)
Puerto Rico	2061 (1255, 3322)	52.8 (32.2, 85.4)	1473 (919, 2349)	59.2 (36.8, 94.2)	12.1 (3.2, 20.1)
Saint Kitts and Nevis	25 (16, 41)	51.4 (31.5, 82.5)	29 (18, 47)	58.2 (36.2, 94.7)	13.3 (4.4, 24.1)
Saint Lucia	86 (54, 139)	49.4 (30.8, 79.8)	82 (50, 133)	55.9 (34, 90.2)	13.3 (4.7, 22.7)
Saint Vincent and the Grenadines	70 (43, 111)	48.4 (29.7, 77.3)	58 (36, 93)	54.8 (34.3, 87.8)	13.1 (3.7, 23.6)
Suriname	230 (142, 369)	49.5 (30.7, 79.5)	309 (189, 490)	54.7 (33.5, 86.9)	10.7 (0.9, 20.8)
Trinidad and Tobago	701 (433, 1123)	51.6 (31.9, 82.6)	660 (410, 1047)	57.4 (35.5, 91.8)	11.3 (3.9, 20.4)
United States Virgin Islands	61 (37, 100)	53.4 (32.7, 87.6)	35 (22, 55)	59.3 (36.7, 92.7)	11 (2.2, 19.7)
Armenia	1229 (753, 1968)	35 (21.4, 56.1)	894 (548, 1416)	38.8 (23.7, 61.7)	11 (1.5, 21)
Azerbaijan	3055 (1862, 4783)	36.6 (22.3, 57.3)	3635 (2235, 5806)	39.5 (24.1, 63.3)	7.7 (−1.8, 18.8)
Georgia	1949 (1187, 3119)	37.2 (22.6, 59.7)	1012 (612, 1593)	38.9 (23.4, 61.2)	4.5 (−4.7, 14.9)
Kazakhstan	6352 (3880, 10 101)	36.3 (22.2, 57.8)	6711 (4111, 10 871)	39.8 (24.5, 64.8)	9.6 (0.8, 19.8)
Kyrgyzstan	1815 (1116, 2900)	35.8 (22.1, 57.2)	2688 (1667, 4282)	38.4 (23.8, 61.2)	7.2 (−2.3, 17.5)
Mongolia	939 (575, 1484)	34.6 (21.2, 54.3)	1178 (712, 1870)	38.7 (23.3, 61.3)	11.7 (1.8, 22.2)
Tajikistan	2232 (1366, 3549)	35 (21.4, 55.4)	4091 (2572, 6473)	37.6 (23.7, 59.6)	7.6 (−2.6, 18.9)
Turkmenistan	1609 (994, 2552)	36.2 (22.4, 57.2)	2110 (1297, 3366)	39.6 (24.4, 63.2)	9.5 (−1.2,20)
Uzbekistan	8731 (5327, 14 111)	35.3 (21.7, 57)	12 789 (7922, 20 369)	39.4 (24.4, 62.7)	11.7 (0.9, 22.1)
Albania	1120 (687, 1779)	29.1 (17.9, 46.1)	709 (440, 1126)	32.6 (20,51.9)	12.2 (2.6,23.4)
Bosnia and Herzegovina	1330 (822, 2134)	28.6 (17.7, 46)	762 (462, 1215)	33.4 (20,53.3)	16.4 (6.1,28.9)
Bulgaria	2353 (1461, 3795)	31 (19.3, 50.1)	1491 (932, 2373)	35.3 (22.1, 56.2)	13.6 (3.7, 24.8)
Croatia	1261 (795, 1959)	29.6 (18.7, 46.1)	922 (572, 1455)	33 (20.5, 52.1)	11.5 (1.1, 20.9)
Czechia	2915 (1796, 4555)	29.8 (18.4, 46.5)	2273 (1382, 3630)	33.6 (20.4, 53.9)	12.9 (2.7, 25.6)
Hungary	2764 (1670, 4399)	29.5 (17.8, 46.8)	2107 (1304, 3390)	33.4 (20.6, 53.8)	13.4 (3, 25)
Montenegro	193 (119, 303)	30.2 (18.7, 47.5)	166 (104, 263)	34 (21.3, 54)	12.4 (2.8, 22.7)
North Macedonia	599 (372, 962)	29.3 (18.2, 47)	522 (321, 839)	32.9 (20.2, 52.6)	12.4 (2, 23.6)
Poland	11 586 (7247, 18 245)	32.9 (20.6, 51.8)	8655 (5379, 13 720)	34.3 (21.3, 54.1)	4.3 (−1, 9.7)
Romania	6492 (3958, 10 360)	28 (17.1, 44.7)	4217 (2651, 6686)	32.2 (20.1, 51.4)	15 (3.4, 27.4)
Serbia	2656 (1668, 4166)	29.7 (18.6, 46.6)	2300 (1444, 3662)	33.6 (21, 53.5)	13 (3.2, 23.8)
Slovakia	1539 (949, 2446)	29.7 (18.3, 47.2)	1215 (744, 1901)	33.6 (20.6, 52.4)	13 (3.1, 24.3)
Slovenia	565 (346, 885)	31.3 (19, 48.8)	439 (273, 688)	34.5 (21.5, 54.1)	10.4 (0.2, 21.2)
Colombia	18 447 (11 387, 30 244)	46.8 (28.9, 76.7)	24 822 (15 230, 39 243)	53.1 (32.7, 84)	13.5 (3.2,24.8)
Costa Rica	1722 (1052, 2720)	48.4 (29.7, 76.5)	2382 (1474, 3786)	54.7 (33.7, 87)	13 (4.3, 22.7)
El Salvador	3213 (1994, 5048)	46.7 (29.1, 73.6)	3609 (2228, 5745)	53.5 (33.1, 85.1)	14.4 (4.5, 23.9)
Guatemala	4708 (2910, 7629)	44.8 (27.8, 72)	9814 (6174, 15 574)	51.8 (32.6, 82.1)	15.5 (6.2, 27.2)
Honduras	2780 (1673, 4393)	46.1 (27.9, 72.7)	6368 (3898, 10 234)	52 (31.9, 83.7)	12.9 (3.4, 22.4)
Mexico	54 912 (33 881, 87 331)	49 (30.2, 77.9)	71 640 (43 983, 113 703)	54.5 (33.5, 86.5)	11.2 (9, 13.6)
Nicaragua	2324 (1400, 3689)	45.6 (27.6, 72.2)	3844 (2379, 6092)	52.2 (32.3, 82.7)	14.5 (5.4, 24.4)
Panama	1481 (899, 2368)	50.9 (30.8, 81.2)	2476 (1515, 4015)	57.5 (35.2, 93.2)	13.1 (4.5, 22.9)
Venezuela (Bolivarian Republic of)	11 534 (7076, 18 534)	49.6 (30.6, 79.6)	13 709 (8514, 22 042)	54.8 (33.9, 88.3)	10.3 (0.7, 19.7)
Angola	7749 (4720, 12 565)	62.8 (38.2, 101.3)	30 433 (18 681, 48 730)	74.6 (45.5, 119)	18.8 (10.7, 29)
Central African Republic	2005 (1201, 3254)	62.1 (37.1, 100.8)	4822 (2972, 7795)	69.2 (42.6, 111.5)	11.4 (2.7, 21.3)
Congo	2073 (1270, 3363)	66.9 (41.1, 107.9)	5031 (3128, 7929)	77.1 (48.1, 121.2)	15.2 (7.9, 23.8)
Democratic Republic of the Congo	28 960 (17 952, 45 422)	63 (39.1, 99)	81 189 (50 481, 129 023)	70.8 (44.2, 112.5)	12.3 (3.4, 22.6)
Equatorial Guinea	311 (189, 489)	62.2 (37.5, 97.5)	1635 (1005, 2591)	78.7 (48.3, 123.9)	26.5 (17.3, 36.3)
Gabon	777 (470, 1227)	66.2 (40.3, 104.8)	1723 (1049, 2741)	79 (48.2, 125.5)	19.4 (10.8, 29.5)
China	917 928 (572 154, 1 443 296)	68.2 (42.5, 107.2)	849 659 (530 715, 1 331 378)	80.7 (50.5, 126.6)	18.4 (16.1, 21)
Democratic People's Republic of Korea	14 537 (9312, 23 061)	68.4 (43.7, 108.8)	17 205 (10 703, 26 900)	76.3 (47.3, 120.2)	11.6 (3.3, 19.5)
Belarus	3325 (2025, 5270)	36.2 (21.9, 57.4)	2482 (1545, 3989)	40.1 (24.9, 65)	11 (0.7, 21.8)
Estonia	495 (307, 782)	36.5 (22.5, 57.7)	354 (221, 570)	40.6 (25.4, 65.6)	11.5 (1.7, 23.1)
Latvia	826 (512, 1316)	36.6 (22.5, 58.5)	488 (304, 784)	40.9 (25.4, 66.1)	11.6 (1.7, 23)
Lithuania	1239 (756, 1950)	36.8 (22.3, 58)	729 (460, 1179)	41.4 (25.9, 66.8)	12.5 (2.3, 23.7)
Republic of Moldova	1518 (935, 2467)	35.9 (22, 58.3)	962 (604, 1559)	39.7 (24.7, 64.6)	10.7 (1.4, 19.6)
Russian Federation	48 916 (30 612, 77 861)	37.1 (23.2, 59)	41 237 (25 850, 65 620)	40.5 (25.2, 64.4)	9.2 (7.2, 11.3)
Ukraine	16 501 (10 072, 26 085)	36.5 (22.3, 57.7)	11 386 (7022, 18 254)	39.5 (24.3, 63.4)	8.1 (−1.2, 18.1)
Burundi	5000 (3112, 7996)	77.2 (48.3, 122.8)	14 313 (8810, 23 052)	85.5 (52.5, 137.5)	10.7 (2.5, 20.3)
Comoros	467 (286, 735)	79.3 (48.7, 124.9)	794 (492, 1231)	92.4 (57.4, 143)	16.5 (8.2, 24.9)
Djibouti	424 (267, 676)	76.1 (47.9, 120.8)	1225 (748, 1962)	87.3 (53.4, 140)	14.8 (7, 23.4)
Eritrea	3291 (2097, 5202)	76.1 (48.9, 119.8)	7182 (4462, 11 203)	89.2 (55.6, 139.3)	17.2 (9.4, 26.2)
Ethiopia	45 690 (28 374, 72 021)	74.1 (46, 117)	124 693 (77 395, 197 908)	86.5 (53.7, 137.4)	16.8 (12.1, 21.2)
Kenya	23 389 (14 563, 37 302)	77.5 (48.3, 123.3)	59 241 (37 024, 93 991)	89.1 (55.7, 141.4)	15 (13.2, 16.8)
Madagascar	11 439 (7033, 18 018)	77.6 (47.8, 121.8)	32 400 (20 167, 51 553)	87.9 (54.8, 139.7)	13.2 (5.7, 21)
Malawi	9101 (5626, 14 327)	76 (47.1, 119.3)	23 938 (14 993, 38 208)	88 (55.2, 140)	15.9 (7.7, 24.3)
Mozambique	11 908 (7376, 18 802)	73.7 (45.8, 115.8)	34 498 (21 608, 54 340)	85.2 (53.5, 134.2)	15.6 (7.7, 24.9)
Rwanda	6948 (4285, 11 029)	79.5 (49.1, 125.1)	15 139 (9309, 23 978)	91 (56, 143.5)	14.5 (6.8, 23.3)
Somalia	6977 (4210, 11 003)	69.8 (42.4, 109.9)	21 025 (13 132, 34 130)	75.3 (47.2, 121.8)	7.9 (−0.5, 17.3)
South Sudan	5711 (3459, 9037)	75.6 (46.1, 120)	10 917 (6859, 17 283)	83.2 (52.5, 131.1)	10 (1.3, 19)
Uganda	15 961 (9943, 25 053)	74 (46.6, 115.7)	51 007 (31 391, 81 020)	88.2 (54.6, 139.2)	19.1 (11, 28.8)
United Republic of Tanzania	25 975 (16 103, 41 347)	79.6 (49.5, 125.9)	66 158 (42 463, 105 568)	89.3 (57.2, 142.1)	12.2 (4.8, 21.3)
Zambia	8254 (5094, 13 120)	78.5 (48.6, 124.7)	23 163 (14 053, 35 903)	91.7 (55.8, 141.6)	16.8 (8.5, 24.7)
Brunei Darussalam	243 (150, 379)	82 (50.8, 127.5)	409 (254, 639)	90.5 (56.4, 141.3)	10.4 (3.2, 17.7)
Japan	101 871 (63 537, 159 052)	86.1 (53.8, 134.2)	76 790 (48 013, 120 388)	93 (58.3, 145.5)	8 (6.3, 10.1)
Republic of Korea	42 952 (26 433, 68 208)	83.1 (51.2, 132.3)	33 473 (20 819, 51 522)	90.8 (56.3, 141.4)	9.3 (2.1, 16.3)
Singapore	2769 (1755, 4341)	82.9 (52.1, 130.2)	3660 (2279, 5778)	91.8 (56.6, 145)	10.7 (3.6, 17.7)
Canada	14 678 (9261, 23 435)	56.4 (35.4, 90.3)	18 147 (11 495, 28 655)	60.9 (38.7, 96.5)	8 (0.6, 16.5)
Greenland	30 (19, 48)	53.6 (33.1, 86.1)	29 (18, 46)	59.2 (36.5, 92.3)	10.6 (3.4, 18.6)
United States of America	147 806 (92 642, 232 946)	61.1 (38.4, 96.4)	188 898 (117 769, 298 843)	66.1 (41.2, 104.4)	8.2 (3.9, 13.6)
Afghanistan	6595 (4103, 10 571)	50.7 (31.7, 81.3)	24 618 (15 262, 39 564)	60.5 (37.6, 97)	19.3 (10.1, 29.5)
Algeria	17 999 (11 073, 28 141)	55.8 (34.4, 87.2)	27 920 (17 486, 43 599)	67.2 (42, 105.3)	20.4 (12.3, 29.9)
Bahrain	299 (184, 464)	57.6 (35.4, 89.6)	956 (582, 1547)	68.8 (42.1, 111.5)	19.3 (9.7, 28.7)
Egypt	39 433 (24 373, 62 114)	59.3 (36.6, 93.1)	81 348 (50 298, 128 751)	69.7 (43.1, 110.2)	17.4 (9.1, 27.1)
Iran (Islamic Republic of)	38 812 (24 073, 61 519)	54.7 (34, 86.8)	50 630 (31 316, 80 185)	67.3 (41.7, 106.6)	22.9 (20.5, 25.4)
Iraq	11 511 (7102, 18 029)	49 (30.4, 76.6)	29 988 (18 163, 48 462)	61.3 (37.2, 99)	25 (14.8, 35.9)
Jordan	2872 (1760, 4688)	56.5 (34.5, 91.3)	9922 (6103, 15 583)	67 (41.3, 105)	18.7 (9.3, 29)
Kuwait	1079 (671, 1725)	59.6 (37.1, 95.7)	2640 (1635, 4107)	71.1 (43.7, 110.6)	19.3 (10, 29.3)
Lebanon	1856 (1132, 2942)	54.9 (33.5, 87)	3358 (2086, 5347)	66.8 (41.6, 106.5)	21.7 (12.6, 31.5)
Libya	3084 (1894, 4916)	54.9 (33.9, 86.8)	4800 (2949, 7681)	67.9 (41.6, 108.5)	23.6 (14.1, 33.3)
Morocco	16 589 (10 172, 26 173)	53.3 (32.8, 84.2)	23 682 (14 703, 37 332)	63.3 (39.3, 99.8)	18.6 (9.4, 27.6)
Oman	1211 (742, 1956)	56.1 (34.2, 90.9)	2864 (1790, 4541)	70.1 (43.8, 111.7)	24.9 (15.8, 35)
Palestine	1372 (856, 2201)	53.3 (33.2, 85.6)	4132 (2501, 6716)	64.8 (39.4, 105.5)	21.7 (12.4, 31.9)
Qatar	244 (151, 385)	58.7 (35.9, 93.3)	1481 (912, 2314)	70.4 (43.3, 110.4)	19.9 (10.4, 29.5)
Saudi Arabia	11 242 (6871, 17 941)	57.9 (35.5, 92)	24 825 (15 144, 39 298)	71.8 (43.4, 114.7)	24.1 (14.8, 33.2)
Sudan	12 670 (7860, 20 029)	51 (31.7, 80.6)	35 401 (21 694, 55 551)	64 (39.1, 100.5)	25.4 (16.3, 35.9)
Syrian Arab Republic	8771 (5427, 14 103)	53.1 (33, 85.6)	11 635 (7187, 18 167)	64.9 (40.3, 100.8)	22.3 (13, 32.7)
Tunisia	5667 (3466, 9084)	55.3 (33.9, 88.7)	6953 (4304, 10 988)	66.7 (41.4, 105.7)	20.6 (11.9, 31.9)
United Arab Emirates	1033 (640, 1625)	59.1 (36.7, 93.8)	4219 (2627, 6805)	72.1 (44.3, 116.8)	21.9 (13.8, 31.2)
Yemen	8150 (5038, 13 228)	49.6 (31, 80.6)	26 574 (16 101, 41 513)	61.6 (37.4, 96.1)	24.1 (14.2, 35)
American Samoa	34 (21, 54)	59.3 (36.7, 94.4)	36 (22, 58)	65.2 (39.6, 104)	9.9 (1.7, 18.3)
Cook Islands	13 (8, 22)	59 (35.9, 95.5)	11 (7, 17)	66.3 (40.8, 106.4)	12.4 (3.6, 20.9)
Fiji	517 (318, 832)	56.4 (34.6, 90.6)	607 (371, 973)	63.6 (38.9, 101.9)	12.8 (4.4, 21.3)
Guam	90 (55, 144)	60.2 (37.1, 96.8)	97 (60, 155)	67.1 (41.7, 107.7)	11.4 (4, 19.8)
Kiribati	45 (27, 71)	52.9 (32.3, 83.7)	81 (50, 128)	58.3 (35.9, 92.4)	10.2 (2.3, 18.7)
Marshall Islands	32 (20, 52)	54.6 (33.7, 87.9)	40 (25, 63)	61.3 (38.4, 97.7)	12.2 (4.8, 21.1)
Micronesia (Federated States of)	74 (45, 120)	55.8 (34.1, 89.5)	74 (46, 117)	61.7 (38.6, 97.1)	10.7 (3, 19.2)
Nauru	7 (4, 10)	56.7 (35.7, 89.5)	8 (5, 13)	62.9 (38.9, 100.4)	10.9 (2.5, 18.7)
Niue	1 (1, 2)	57.6 (36.2, 90)	1 (1, 2)	64.7 (39.5, 102.1)	12.4 (4.3, 21.1)
Northern Mariana Islands	31 (19, 49)	62.1 (38, 99.7)	31 (19, 48)	66.3 (41.5, 104.3)	6.8 (0.1, 14.9)
Palau	11 (7, 17)	59.5 (36.8, 96)	10 (6, 15)	65.6 (40.2 102.9)	10.3 (2.2, 18.6)
Papua New Guinea	2575 (1570, 4132)	51.6 (31.3, 82.7)	6757 (4197, 10 817)	57.2 (35.5,91.2)	10.9 (2.6, 19.9)
Samoa	126 (77, 203)	56.6 (34.8, 91.6)	152 (94, 244)	62.3 (38.6, 100.5)	10.2 (1.7, 18.9)
Solomon Islands	226 (137, 362)	51.7 (31.5, 82.5)	470 (291, 746)	57.7 (35.7, 91.5)	11.6 (2.7, 20.6)
Tokelau	1 (1, 2)	55.1 (33.7, 88.4)	1 (1, 1)	62.7 (38.8, 100.1)	13.7 (4.8, 22.5)
Tonga	72 (43, 114)	56.6 (34, 90.4)	76 (48, 124)	63.3 (39.9, 102.6)	11.8 (4, 20.1)
Tuvalu	5 (3, 8)	54.8 (33.3, 87)	8 (5, 13)	61.6 (37.7, 99.2)	12.4 (4.7,20.9)
Vanuatu	97 (61, 155)	53.2 (33.5, 84.7)	215 (131, 341)	59.5 (36.2, 93.9)	11.7 (3.7, 20.1)
Bangladesh	54 487 (33 155, 86 301)	41.2 (25.2, 65.2)	88 318 (53 643, 136 950)	49 (29.7, 76)	18.8 (8, 29.5)
Bhutan	334 (206, 544)	39.1 (24, 63.9)	379 (238, 603)	46.6 (29.1, 74.1)	19.2 (8.1, 30.3)
India	420 583 (262 231, 668 300)	42.5 (26.5, 67.5)	799 135 (500 615, 1 270 272)	51 (31.9, 81.2)	20 (17.7, 22.7)
Nepal	8614 (5297, 13 890)	37.8 (23.4, 60.6)	16 128 (9977, 26 700)	44.3 (27.5, 73.1)	17.1 (7.8, 27.8)
Pakistan	51 188 (32 169, 81 941)	37.9 (23.7, 60.7)	125 397 (76 906, 203 051)	44.8 (27.5, 72.5)	18.3 (12, 24.7)
Cambodia	7127 (4421, 11 478)	58.3 (36.5, 93.5)	12 192 (7590, 19 403)	66.5 (41.4, 105.8)	14 (5.3, 22.9)
Indonesia	143 512 (89 153, 229 028)	63.2 (39.3, 100.6)	201 756 (125 776, 320 447)	72.7 (45.4, 115.7)	15.1 (12.6, 17.5)
Lao People's Democratic Republic	2853 (1750, 4565)	57.5 (35.1, 91.5)	5398 (3355, 8612)	66.2 (41.1, 105.7)	15.2 (6.5, 24.4)
Malaysia	13 100 (8165, 20 803)	63.2 (39.4, 100.2)	23 208 (14 409, 37 108)	71.9 (44.7, 115.2)	13.8 (5.9, 22.2)
Maldives	154 (96, 241)	57 (35.6, 89)	305 (190, 492)	67.4 (42.1, 109.5)	18.3 (8.8, 28.3)
Mauritius	782 (497, 1273)	62.3 (39.5, 101.4)	768 (480, 1226)	72 (44.7, 114.9)	15.5 (6.8, 24.5)
Myanmar	29 018 (18 440, 45 653)	58.6 (37.2, 92.3)	40 691 (25 229, 64 978)	68.2 (42.2, 108.9)	16.4 (8.7, 24.2)
Philippines	48 551 (30 401, 76 410)	62.5 (39.1, 98.2)	88 232 (55 491, 139 844)	69.9 (43.9, 110.8)	11.7 (10.1, 13.3)
Seychelles	56 (34, 91)	65.6 (40.4, 106.2)	67 (42, 105)	72.6 (44.9, 114.2)	10.6 (3.6, 18.9)
Sri Lanka	12 407 (7594, 20 075)	61.9 (38, 100.1)	14 715 (9148, 23 354)	69.5 (43.2, 110.2)	12.2 (3.6, 20.3)
Thailand	40 539 (25 167, 64 645)	59.6 (37.1, 95.3)	32 711 (19 895, 52 121)	65.6 (39.6, 104.5)	10 (2.1, 19.4)
Timor‐Leste	512 (316, 808)	58 (35.9, 91.6)	1222 (754, 1972)	69.2 (42.9, 111.4)	19.4 (10.7, 27.7)
Viet Nam	50 281 (30 905, 78 881)	61.1 (37.6, 95.7)	63 575 (39 908, 101 338)	70.2 (44.1, 111.9)	14.9 (7, 23.4)
Uruguay	2091 (1290, 3292)	68.2 (42, 107.3)	2241 (1411, 3509)	73.7 (46.2, 115.3)	8.1 (1.3, 16.1)
Argentina	23 038 (14 387, 36 833)	67.8 (42.4, 108.1)	32 542 (20 068, 51 359)	73.7 (45.4, 116.5)	8.7 (1.2, 16.4)
Chile	9958 (6229, 15 607)	67.9 (42.8, 106.4)	12 564 (7849, 19 712)	74.1 (46.2, 116.9)	9.1 (1.6, 16.8)
Botswana	1153 (698, 1823)	66.9 (40.9, 105.9)	2037 (1259, 3251)	77.8 (48, 124.1)	16.2 (8.1, 24.8)
Eswatini	682 (419, 1117)	64.9 (39.9, 105.8)	1069 (663, 1713)	75.1 (46.7, 120.4)	15.7 (7.1, 24.4)
Lesotho	1204 (738, 1947)	64.7 (39.8, 104.3)	1759 (1073, 2767)	74.8 (45.7, 117.6)	15.5 (6.8, 24.8)
Namibia	1211 (728, 1967)	67.1 (40.7, 108.6)	2251 (1358, 3653)	77.7 (47, 126.1)	15.9 (8, 24.7)
South Africa	31 443 (19 547, 50 274)	69.2 (43, 110.5)	45 313 (28 110, 71 386)	78 (48.4, 122.9)	12.7 (9.6, 16.3)
Zimbabwe	9079 (5544, 14 480)	66.4 (40.7, 105)	14 408 (8773, 23 226)	73.4 (44.7, 117.8)	10.6 (2.8, 19.7)
Brazil	81 641 (50 877, 128 920)	45 (28, 71.1)	96 464 (59 955, 153 118)	49.9 (30.9, 79.2)	10.8 (8.2, 13.5)
Paraguay	2277 (1376, 3535)	47.1 (28.5, 73.2)	4170 (2521, 6620)	53.4 (32.3, 84.7)	13.4 (3.5, 25.3)
Andorra	55 (35, 86)	102.7 (64.8, 158.6)	69 (44, 109)	110.1 (70, 174.7)	7.2 (−0.1, 14.5)
Austria	6956 (4337, 10 910)	100.5 (62.4, 158.1)	7114 (4474, 11 095)	107.8 (67.4, 169.3)	7.2 (1.1, 14.6)
Belgium	8700 (5444, 13 813)	99.6 (62.2, 158.3)	9455 (5911, 14 621)	107.9 (67.8, 167.2)	8.4 (2.1, 14.9)
Cyprus	757 (480, 1173)	98.1 (62.2, 152.3)	1170 (743, 1831)	109.9 (69.2, 173)	12.1 (5.4, 19.5)
Denmark	4784 (3004, 7425)	102.8 (64.4, 160.5)	5056 (3201,7954)	110.1 (69.1174)	7.1 (0.6,13.7)
Finland	4353 (2699, 6910)	101.4 (62.8, 160.6)	4535 (2821, 7183)	109.5 (68.4, 174)	7.9 (2, 15.1)
France	50 364 (31 582, 78 021)	94.3 (59.3, 146.4)	53 545 (33 226, 83 343)	102.1 (63, 159.9)	8.3 (1.4, 15.7)
Germany	76 354 (47 731, 120 237)	115.1 (71.7, 181.9)	72 242 (44 742, 113 576)	121 (75.3, 191.6)	5.2 (−0.8, 11.4)
Greece	9077 (5646, 14 076)	94.1 (58.4, 145.8)	7391 (4694, 11 582)	103.6 (64.8, 163.5)	10.2 (3.1, 17.9)
Iceland	256 (159, 400)	99.6 (62, 155.9)	321 (201, 502)	108.8 (68.4, 170.5)	9.2 (2.6, 16.3)
Ireland	3822 (2438, 5835)	99.5 (63.7, 151.9)	4599 (2872, 7219)	109.7 (68.3, 173.4)	10.2 (3.7, 16.9)
Israel	5293 (3260, 8328)	99.8 (61.7, 156.4)	9735 (6101, 15 356)	107.9 (67.6, 170.1)	8.1 (1.5, 14.6)
Italy	53 050 (33 328, 82 473)	100.9 (63.3, 157.2)	43 804 (27 448, 68 932)	107.7 (67.6, 170)	6.8 (4.6, 9.2)
Luxembourg	341 (211, 543)	105.1 (65, 166.2)	572 (365, 896)	113.5 (72.3, 177.9)	8 (1.6, 14.5)
Malta	336 (211, 519)	96 (60.1, 148.1)	319 (200, 498)	104.5 (64.5, 163.7)	8.9 (2.2, 15.9)
Monaco	21 (13, 34)	103 (64.4, 163.5)	27 (17, 42)	109.5 (67.2, 173.4)	6.2 (0.5, 12.5)
Netherlands	14 193 (8980, 21 929)	101.4 (63.8, 157.5)	14 543 (9218, 22 933)	109.6 (69.4, 173.8)	8 (1.3, 15.2)
Norway	3982 (2490, 6170)	103.1 (64.6, 159.8)	4891 (3083, 7682)	112.3 (70.8, 177.1)	8.9 (5.9, 11.8)
Portugal	10 750 (6651, 17 182)	106.5 (65.9, 170.3)	8830 (5554, 13 880)	116.3 (73.7, 182.3)	9.2 (2.6, 16.4)
San Marino	24 (15, 37)	103.3 (63.7, 161.2)	27 (17, 42)	110.8 (69.2, 173)	7.3 (0.8, 14.1)
Spain	38 165 (23 446, 58 964)	97.8 (60.1, 151.1)	34 497 (21 760, 54 242)	106 (66.5, 166.4)	8.3 (1.8, 15.4)
Sweden	6786 (4263, 10 582)	94.9 (59.7, 147.8)	8153 (5106, 12 737)	101.9 (63.9, 159.2)	7.4 (2.2, 12.8)
Switzerland	6159 (3797, 9750)	104.1 (64.1, 163.4)	7077 (4444, 11 034)	109.7 (68.8, 172.4)	5.3 (−0.9, 12.8)
United Kingdom	50 761 (31 963, 79 137)	101 (63.6, 157.9)	58 857 (36 977, 92 375)	110 (69.3, 172.7)	8.9 (7.4, 10.6)
Benin	3271 (2004, 5281)	58.7 (36, 94.9)	11 362 (7011, 18 017)	67.6 (42, 106.7)	15.1 (6.6, 24.5)
Burkina Faso	6388 (3959, 9995)	56.2 (34.5, 88)	18 443 (11 391, 29 777)	65.4 (40.6, 105.5)	16.5 (8.7, 25.8)
Cabo Verde	258 (160, 407)	59.6 (36.9, 94.4)	424 (264, 657)	70.9 (44.3, 109.9)	19.1 (11, 27.9)
Cameroon	7525 (4623, 12 188)	60.6 (37.4, 97.6)	29 014 (17 796, 46 017)	72.4 (44.4, 114.5)	19.3 (10.5, 28.7)
Chad	3966 (2441, 6218)	56.1 (34.7, 88.2)	14 367 (8920, 22 712)	63.8 (39.6, 101)	13.7 (4.7, 23)
Gambia	686 (423, 1104)	57.2 (35.2, 92)	2097 (1271, 3384)	67.2 (40.7, 108)	17.5 (8.8, 27.2)
Ghana	10 516 (6542, 16 837)	57.9 (35.9, 92.7)	27 786 (17 110, 45 159)	68.3 (42, 110.7)	18 (9.9, 28)
Guinea	3771 (2315, 6003)	57.5 (35.2, 91.6)	11 155 (6847, 17 776)	66.5 (40.9, 105.5)	15.6 (7, 25.6)
Guinea‐Bissau	733 (455, 1164)	58.8 (36.9, 93.1)	1763 (1101, 2768)	67.8 (42.4, 106.3)	15.2 (6.5, 24.4)
Liberia	1621 (1029, 2587)	57.3 (36.2, 91.2)	4610 (2769, 7346)	66 (39.8, 105)	15.3 (6.9, 24.9)
Mali	5561 (3403, 8758)	56.2 (34.6, 88.2)	19 753 (12 365, 31 222)	64.4 (40.3, 101.9)	14.6 (6, 23.7)
Mauritania	1467 (894, 2348)	59.9 (36.5, 95.9)	3856 (2388, 6238)	69.5 (43, 112.2)	16.1 (7.7,25.4)
Niger	5231 (3207, 8163)	55.1 (33.9, 86)	19 969 (12 501, 32 079)	61.5 (38.5, 98.2)	11.7 (2.1, 22.4)
Nigeria	68 356 (42 524, 107 480)	63.2 (39.3, 99.4)	219 259 (136 453, 345 478)	73.4 (45.7, 115.6)	16.1 (14.3, 18.2)
Sao Tome and Principe	93 (57, 152)	60.3 (37.1, 97.5)	194 (120, 310)	70.9 (43.7, 112.8)	17.5 (8.4, 26.8)
Senegal	5442 (3326, 8711)	58.4 (35.7, 93.1)	13 635 (8401, 21 929)	67.8 (41.6, 108.6)	16 (5.9, 26.2)
Sierra Leone	2749 (1707, 4446)	58.7 (36.7, 94.8)	7513 (4709, 12 043)	67.4 (42.3, 108.2)	15 (7, 24.3)
Togo	2740 (1697, 4323)	59.9 (37.1, 94.6)	6989 (4243, 11 035)	68.6 (41.8, 108.3)	14.6 (6.7, 23.6)
Taiwan	15 574 (9709, 24 607)	69.6 (43.4, 110)	12 772 (7952, 19 940)	81.6 (50.4, 129.4)	17.4 (10.2, 25.6)
Côte d'Ivoire	8611 (5358, 13 577)	58.9 (36.6, 92.7)	22 858 (14 201, 37 023)	68.3 (42.5, 110.6)	15.9 (7.7, 25.9)
Turkey	39 839 (24 244, 63 037)	56.1 (34.3, 88.7)	52 985 (31 803, 83 359)	68.6 (41.1, 108.1)	22.2 (14, 32.1)

### 3.4 Age and Gender Patterns

**FIGURE 3:**
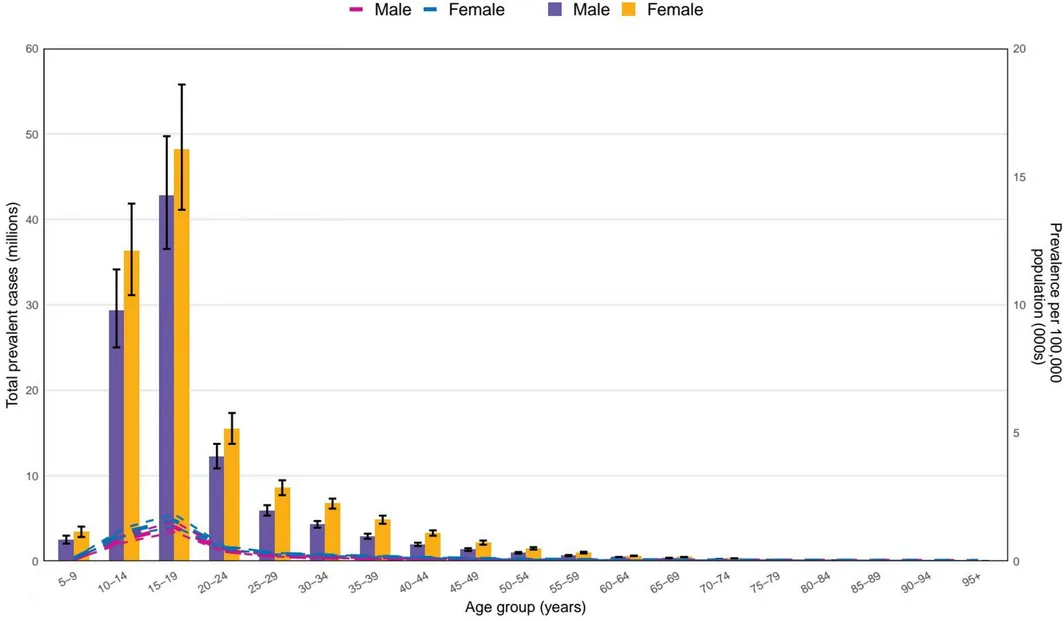
Global prevalence cases and acne vulgaris incidence per 100 000 population by age and sex in 2021. Lines represent prevalence cases for males and females, with 95% uncertainty intervals (based on https://ghdx.healthdata.org/gbd‐results‐tool).

**FIGURE 4:**
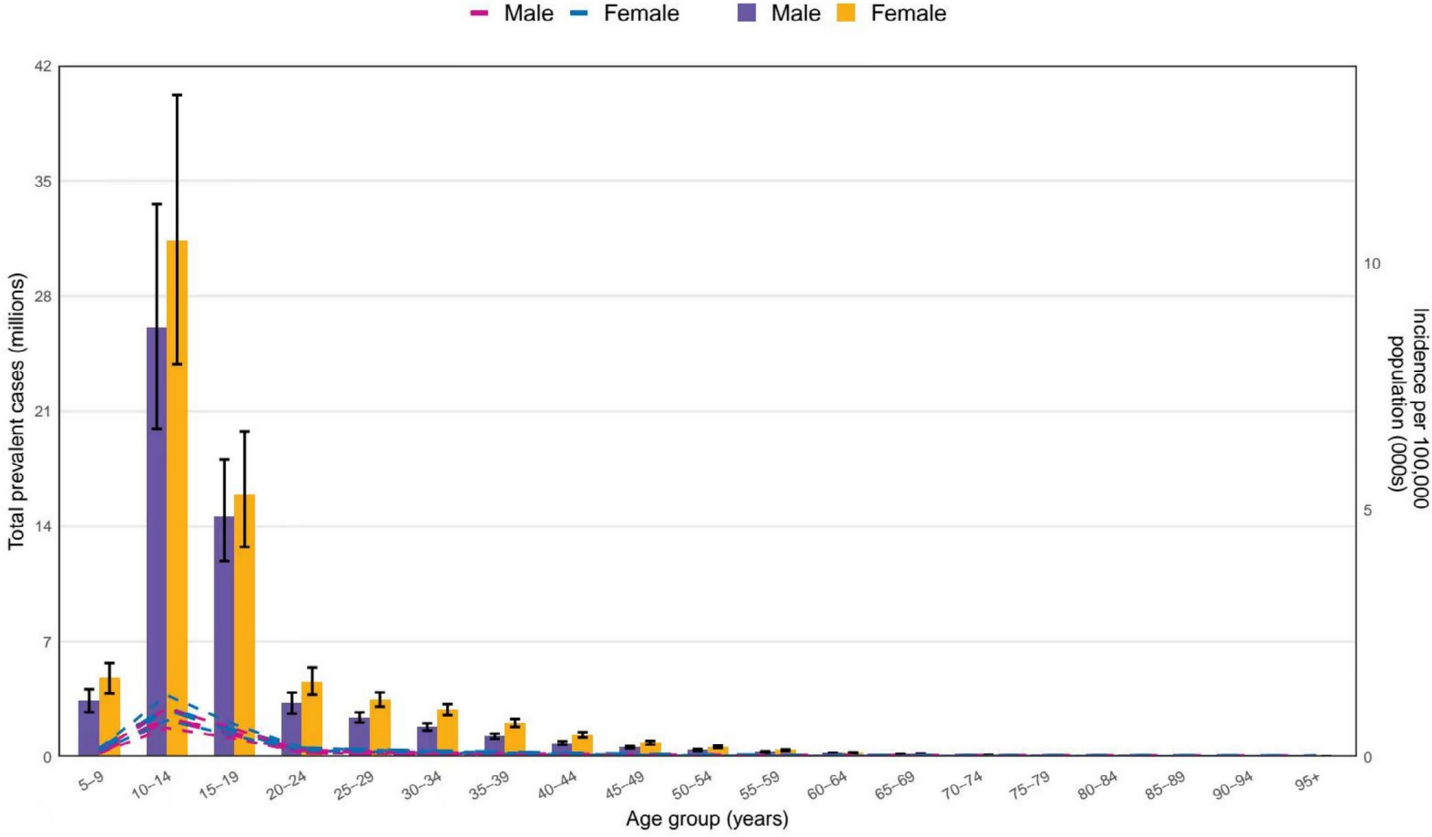
Global incidence cases and incidence rate of acne vulgaris per 100 000 population by age and sex in 2021. Lines represent incidence cases for males and females with 95% uncertainty intervals (based on https://ghdx.healthdata.org/gbd‐results‐tool).

**FIGURE 5:**
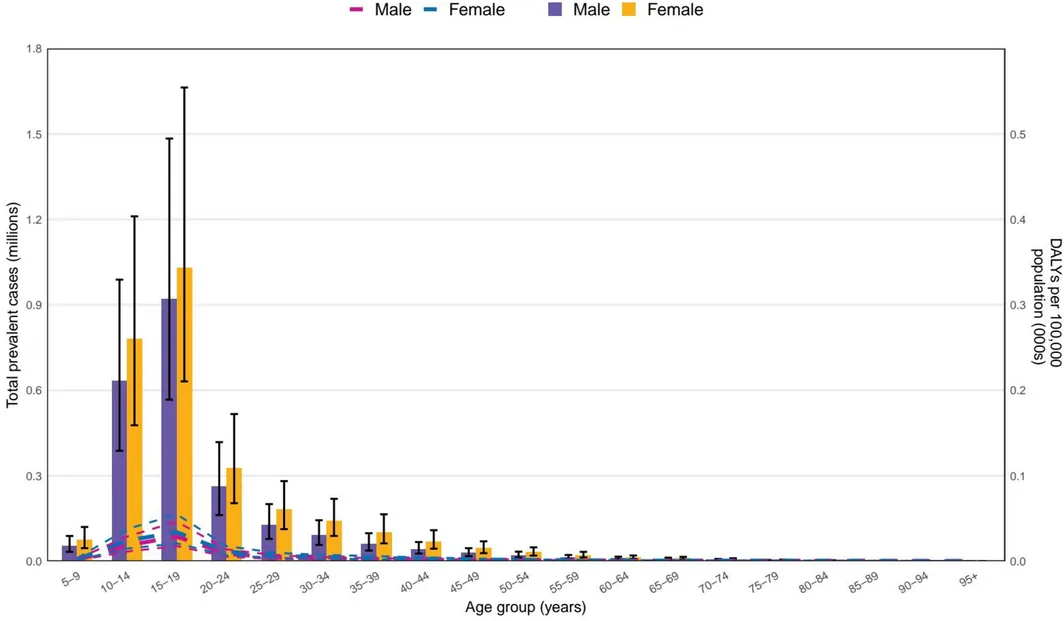
Global DALYs and DALY rate per 100 000 population for acne vulgaris in 2021, by age and sex. Lines represent DALYs cases for males and females, with 95% uncertainty intervals (based on https://ghdx.healthdata.org/gbd‐results‐tool).

In 2021, the age‐standardized prevalence of acne vulgaris worldwide peaked in the 15–19 age group and remained elevated throughout adolescence, declining significantly with increasing age thereafter. Similarly, the number of cases was most concentrated in the 15–19 age group (Figure 3). In 2021, the age‐standardized incidence rate of acne vulgaris globally peaked in the 10–14 age group, with females exhibiting higher incidence than males across all adolescent age groups. Regardless of sex, the highest incidence occurred in the 10–14 age group, followed by a rapid decline with increasing age (Figure 4). The global DALY rate for acne vulgaris peaked in the 15–19 age group before gradually declining, with a steeper decline among females than males. Additionally, the absolute number of DALYs reached its highest level in the 15–19 age group, being more pronounced among females in this cohort (Figure 5).

### 3.5 Association With Socio‐Demographic Index

**FIGURE 6:**
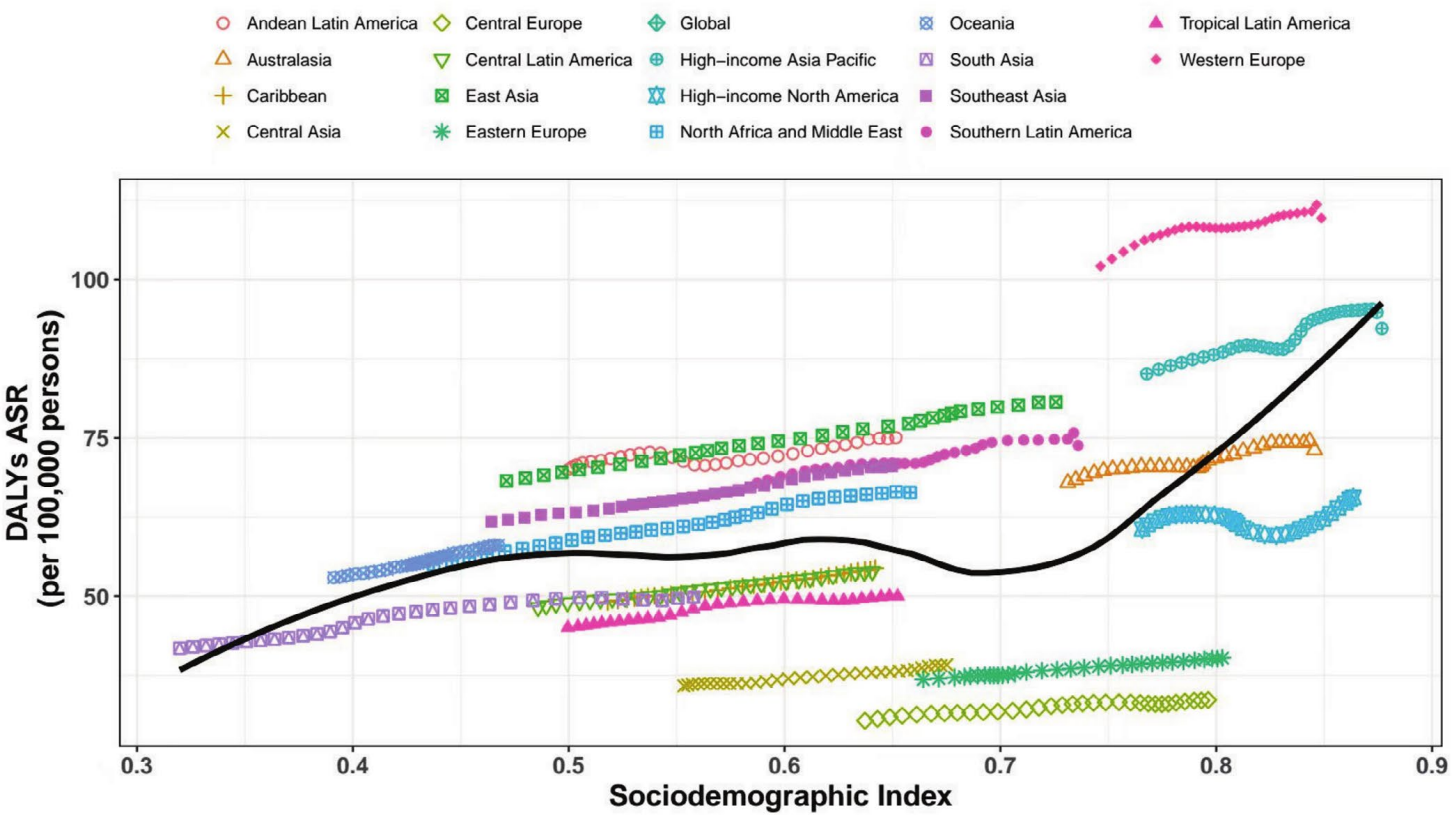
Age‐standardized disability‐adjusted life year (DALY) rates for acne vulgaris by sociodemographic index across 21 global disease burden regions, 1990–2021. Each region displays 30 points illustrating observed age‐standardized DALY rates from 1990 to 2021. The solid line represents the expected value based on the sociodemographic index and disease incidence across all regions. Regions above the solid line indicate a burden exceeding expectations, while those below indicate a burden below expectations (based on https://ghdx.healthdata.org/gbd‐results‐tool).

At the regional level, our analysis of data from 1990 to 2021 indicates an overall positive correlation between age‐standardized acne vulgaris DALY rates and the Socio‐Demographic Index (SDI). Age‐standardized DALY rates increase with rising SDI and peak in high‐SDI regions. Specifically, this association pattern exhibits significant geographic heterogeneity. Regions with high SDI, particularly the Asia‐Pacific high‐income region and Western Europe, bear the highest burden of acne vulgaris, with age‐standardized DALY rates exceeding the global average. Conversely, low‐SDI regions, such as parts of South Asia, exhibit relatively lower disease burdens. Some middle‐to‐high SDI regions, including Central Europe, Eastern Europe, and Central Asia, show low disease burden rates. Based on sociodemographic regions, Oceania, North Africa and the Middle East, Southeast Asia, Andean Latin America, Southern Latin America, East Asia, Asia‐Pacific High‐Income, and Western Europe exhibited DALY rates above expectations. Conversely, South Asia, Central Latin America, the Caribbean, Tropical Latin America, High‐Income North America, Central Europe, Eastern Europe, and Central Asia recorded DALY rates below expectations (Figure 6).

**FIGURE 7:**
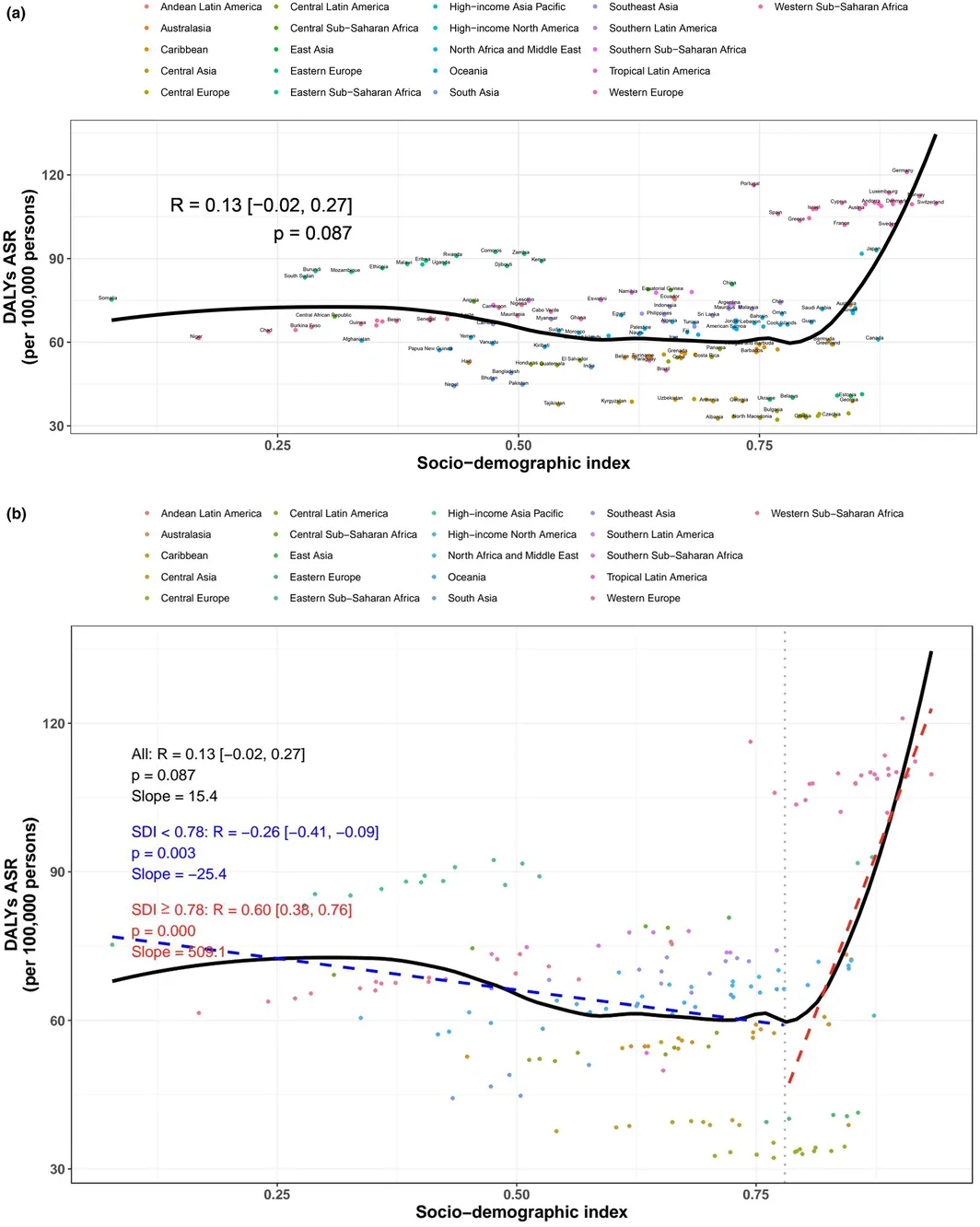
(a) Age‐standardized disability‐adjusted life year (DALY) rates for acne vulgaris across 204 countries by sociodemographic index, 1990–2021. Expected values based on all regions' sociodemographic indices and disease incidence are shown as solid lines. Regions above the solid line indicate higher‐than‐expected burden, while those below indicate lower‐than‐expected burden (based on https://ghdx.healthdata.org/gbd‐results‐tool). (b) Age‐standardized disability‐adjusted life year (DALY) rates for acne vulgaris across 204 countries by sociodemographic index, 1990–2021. Expected values based on all regions' sociodemographic indices and disease incidence are shown as solid lines. Added piecewise linear regression analysis before and after the 0.78 threshold. Regions above the solid line indicate higher‐than‐expected burden, while those below indicate lower‐than‐expected burden (based on https://ghdx.healthdata.org/gbd‐results‐tool).

At the national level, the association between acne burden and the Socio‐demographic Index (SDI) from 1990 to 2021 did not reach statistical significance (*R* = 0.13, *p* = 0.087), suggesting complex underlying patterns. Piecewise linear regression analysis identified a critical threshold at SDI = 0.78. In the low‐to‐middle SDI group (SDI < 0.78), the relationship was inversely correlated (*R* = −0.26, *p* = 0.003), indicating that higher social development levels were linked to reduced disease burden. Conversely, in the higher SDI group (SDI ≥ 0.78), this association reversed, showing a strong positive correlation (*R* = 0.60, *p* < 0.001). The significant change in slope (interaction term in piecewise regression, *p* < 0.05) confirms a nonlinear epidemiological transition at this developmental threshold. However, high‐income Western European countries such as Portugal, Spain, and France, along with Israel, exhibited disease burdens significantly higher than expected for their SDI levels. Conversely, several Central Asian countries with medium‐to‐high SDI, as well as low‐SDI nations like Niger, Chad, and Papua New Guinea, showed burdens substantially lower than projected (Figure 7).

### 3.6 Projected Trends in the Disease Burden of Acne Vulgaris in China Over the Next 15 Years

**FIGURE 8:**
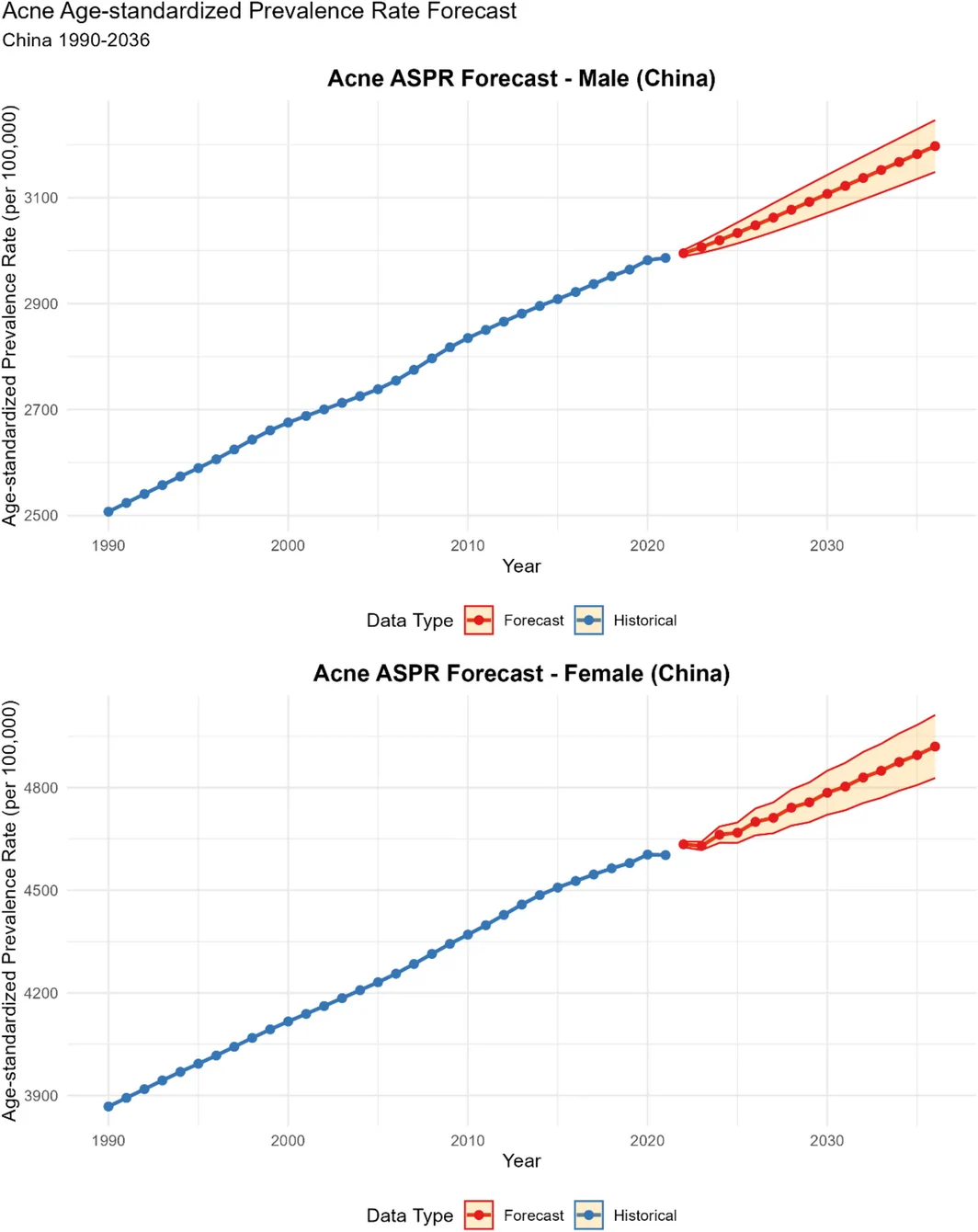
Trends in Acne vulgaris Incidence in China, 1990–2021, and Projected Trends for 2022–2036 (based on https://ghdx.healthdata.org/gbd‐results‐tool).

A predictive model based on observed data from 1990 to 2021 indicates that over the next 15 years (2022–2036), the age‐standardized prevalence of acne vulgaris in China is projected to show a sustained upward trend across both genders. However, a notable feature of this trend is the presence of persistent gender disparities. The male prevalence is projected to rise steadily from approximately 3000 cases per 100 000 population in 2021 to around 3100 cases per 100 000 by 2030, with further increases anticipated by 2036. In contrast, the disease burden among females is markedly higher and exhibits more pronounced growth; projections indicate its prevalence will rise from approximately 4600 cases per 100 000 people in 2021 to over 4800 cases per 100 000 by 2036. These findings underscore that acne vulgaris will remain an increasingly severe public health issue in China, with the disease burden increasingly concentrated among the female population (Figure 8).

## 4 Discussion

This study, based on GBD 2021 data, provides the first comprehensive and systematic analysis of the global disease burden of acne vulgaris from 1990 to 2021. Core findings indicate that acne vulgaris represents a persistently growing global public health issue. Compared to 1990, 2021 saw increases in age‐standardized prevalence rates, incidence rates, and DALY rates for acne vulgaris worldwide (rising by 12.2%, 14.0%, and 12.3%, respectively), with absolute case numbers reaching hundreds of millions. This may result from population growth, improved diagnostic rates, and changes in socioeconomic factors. This trend contrasts with the declining burden of many chronic noncommunicable diseases, highlighting the increasing importance of skin conditions in the global health agenda [15].

The distribution of acne vulgaris burden exhibits marked geographic heterogeneity. Western Europe, high‐income Asia‐Pacific regions, and eastern sub‐Saharan Africa bear the highest disease burden, with age‐standardized DALY rates far exceeding the global average. This distribution pattern may be driven by multiple factors: first, greater healthcare accessibility, diagnostic awareness, and emphasis on personal appearance may lead to more complete case reporting [17, 18, 19]; Lifestyle factors (such as high‐sugar, high‐fat diets), environmental factors, and potentially higher psychological stress levels may also be significant contributors. Numerous studies have documented a clear association between Western diets and the presence and exacerbation of acne vulgaris, with foods like chocolate, French fries, cola, pizza, and dried snacks most frequently linked to acne [20]. Conversely, regions such as Central Europe, Central Asia, and Eastern Europe exhibit lower disease burdens. The specific reasons for this disparity require further investigation but may be related to genetic background, dietary habits, or differences in healthcare systems [21].

At the national level, the burden is particularly pronounced in countries such as Germany and Portugal, while Sudan and Iraq exhibit the highest growth rates, indicating that the burden of acne vulgaris is expanding beyond traditionally recognized high‐income nations to more regions. This shift may be closely linked to globalization, lifestyle changes, and psychological anxiety and depression [22, 23].

This study reaffirms the age and gender disparities in the burden of acne vulgaris. The peak burden is concentrated among adolescents aged 10–19, aligning closely with the pathophysiological mechanism where hormonal changes during puberty activate sebaceous gland secretion [1]. More importantly, the study found that women exhibit higher age‐standardized prevalence, incidence, and DALY rates than men across all age groups, particularly in adulthood—a conclusion consistent with prior research [20]. This reflects the substantial burden of adult acne vulgaris, particularly among women, potentially linked to hormonal fluctuations (e.g., menstrual cycles, polycystic ovary syndrome, pregnancy), cosmetic use, and greater willingness to seek medical care [20, 24, 25]. The psychosocial impact of acne vulgaris on women—including anxiety, depression, and social avoidance—may further exacerbate its disease burden, resulting in a flatter decline in DALY rates with age among women compared to men [24].

The relationship between acne vulgaris burden and SDI is not a simple linear positive correlation but exhibits a complex “upward‐sloping curve.” In low‐SDI regions, the burden initially rises with socioeconomic development before plateauing or even slightly declining. However, once SDI exceeds approximately 0.78, the burden shows a rapid upward trend. This nonlinear relationship reveals the profound influence of health's social determinants. In high‐SDI countries, the “manifestation” of burden may stem from robust healthcare surveillance systems and heightened disease awareness. Conversely, in low‐SDI countries, burden may be underestimated due to factors including scarce healthcare resources, underdiagnosis, and the diversion of resources toward more pressing health issues (e.g., infectious diseases) [13, 14]. The burden in certain countries (e.g., Portugal, Spain) far exceeds expectations based on their SDI levels, while others (e.g., some Central Asian nations) fall significantly below projections. This strongly suggests that factors beyond socioeconomic conditions—including genetic susceptibility, specific environmental exposures, cultural practices, and localized health policies—play crucial roles in shaping the burden of acne vulgaris [3].

The in‐depth analysis and projections for China in this study add an important regional perspective. China's current burden of acne vulgaris already exceeds the global average, and projections indicate it will continue to rise over the next 15 years, with a particularly pronounced increase in the female burden. The significant rise in age‐standardized prevalence data for acne vulgaris in China may be associated with changes in lifestyle and diet, as well as increased psychological stress [26]; this trend may also be closely linked to the rapid expansion of the medical aesthetics industry. The observed increase in reported cases reflects heightened public attention to skin aesthetics—including individuals seeking treatment for acne scars and pitting—rather than a sudden surge in actual acne prevalence [27, 28]. This serves as a wake‐up call for China's public health system. With the nation's rapid socioeconomic development and changing lifestyles, the disease burden of acne vulgaris may intensify further. Therefore, targeted national prevention and control strategies are urgently needed, particularly in adolescent health education and providing accessible dermatological services for women.

## 5 Strengths and Limitations of This Study

This study leverages the GBD—an authoritative, standardized, and globally comprehensive data source—enabling highly comparable analyses across countries and time periods. We employed multiple statistical methods, including EAPC and BAPC models, and examined health inequalities through the SDI perspective, enhancing the study's depth and policy relevance.

However, this study also has several limitations. First, GBD data are inherently model‐estimated results, whose accuracy depends on the quality and quantity of input data. In regions with inadequate medical reporting systems, uncertainty intervals are larger, potentially leading to underestimation. Furthermore, underdiagnosis in low‐SDI countries—driven by limited access to dermatological care and low health‐seeking behavior for a non‐fatal condition like acne vulgaris—represents a major alternative explanation for the observed SDI gradient. This case ascertainment bias likely contributes substantially to the wider uncertainty intervals noted above. Second, the GBD database lacks individual‐level risk factor data for acne vulgaris (e.g., detailed dietary patterns, cosmetic usage frequency, genetic markers), precluding direct verification of causal relationships between these factors and disease burden changes. Finally, projections of future trends are extrapolations from historical data, failing to account for potential impacts of breakthrough therapies or major environmental shifts on acne vulgaris incidence.

## 6 Conclusion

Acne vulgaris represents a significant global public health issue with extensive economic costs and psychosocial impacts. Between 1990 and 2021, not only did the age‐standardized prevalence, incidence, and DALY rates of acne vulgaris show an upward trend globally, but the absolute number of cases also increased substantially, reflecting both population growth and an intensifying disease burden. With evolving lifestyles, heightened psychological stress, and increased awareness of medical aesthetics, acne vulgaris is projected to remain a growing challenge, particularly among populations with high sociodemographic indices, adolescents, and women. This knowledge can guide policymakers in developing targeted prevention, treatment, and mental health support strategies to address the escalating demands associated with acne vulgaris.

## Author Contributions

Qian Zhang: conceptualization, data curation, formal analysis, methodology, software, visualization, writing – original draft, investigation, writing – review and editing. Yanyan Feng: conceptualization, resources, supervision, validation, writing – review and editing. Peixuan Wu: methodology, supervision. Chun Yang: supervision, validation. ZiJie He: data curation.

## Funding

The authors have nothing to report.

## Conflicts of Interest

The authors declare no conflicts of interest.

## Data Availability

Data will be made available on request.
